# Spatiotemporal Distribution and Host–Vector Dynamics of Japanese Encephalitis Virus

**DOI:** 10.3390/v17060815

**Published:** 2025-06-04

**Authors:** Qikai Yin, Bin Li, Ruichen Wang, Kai Nie, Shihong Fu, Songtao Xu, Fan Li, Qianqian Cui, Dan Liu, Huanyu Wang, Guodong Liang

**Affiliations:** 1 National Key Laboratory of Intelligent Tracking and Forecasting for Infectious Diseases, National Institute for Viral Disease Control and Prevention, Chinese Center for Disease Control and Prevention, Beijing 102206, China; yinqk@ivdc.chinacdc.cn (Q.Y.); wangrc96@163.com (R.W.); niekai@ivdc.chinacdc.cn (K.N.); shihongfu@hotmail.com (S.F.); xust@ivdc.chinacdc.cn (S.X.); lifan@ivdc.chinacdc.cn (F.L.); cuiqq@ivdc.chinacdc.cn (Q.C.); 2 Taishan District Center for Disease Control and Prevention, Taian 271000, China; 208043044@stu.jmsu.edu.cn; 3 School of Biological Engineering, Bayingol Vocational and Technical College, Korla 841000, China; liud0516@163.com

**Keywords:** Japanese encephalitis virus, diversity, mosquito vectors, host animals

## Abstract

Japanese encephalitis (JE), a mosquito-borne viral disease caused by the Japanese encephalitis virus (JEV), remains a significant public health threat in Asia. Although vaccination programs have successfully reduced the incidence of JE, challenges persist in the adult population, and the emergence of rare JEV genotypes poses additional risks. In this study, a phylogenetic analysis of the whole JEV genome sequence, along with a temporal–spatial analysis of isolates and a host–vector analysis, was used to examine the changes in JEV transmission dynamics before and after 2012. The results revealed persistent differences between the dominant G1 and G3 genotypes, as well as the re-emergence of G4 and G5 genotypes. Although JEV has been detected in non-traditional vectors and atypical mammalian hosts, *Culex tritaeniorhynchus* and pigs remain the primary vector and amplifying host, respectively. These findings underscore the need to enhance existing JEV genotype surveillance while addressing emerging threats from genotype diversity, host expansion, and geographic spread.

## 1. Introduction

Japanese encephalitis (JE) is a central nervous system infectious disease caused by Japanese encephalitis virus (JEV) transmitted through the bite of infected mosquitoes. JEV is the main cause of viral encephalitis in many countries of Asia, with an estimated 100,000 clinical cases every year [1]. Twenty-four countries in the WHO South-East Asia and Western Pacific Regions have endemic JEV transmission, exposing more than 3 billion people to risks of infection, with an annual incidence of approximately 69,000 cases and a mortality rate of 30%; 70% of survivors have neurological sequelae of varying severities. In particular, China has reported more than 2 million cases of JE from 1950 to 2018, with more than 270,000 deaths [2]. JE is a vaccine preventable disease, although in recent years, with the widespread use of JE vaccines in Asia, the number of JE cases has decreased significantly. However, the challenges faced by the control and prevention of JE in adults, and the arbovirus disease caused by the rare genotype JEV is also considered as an emerging infectious disease, which has attracted extensive attention [3].

JEV is a member of the genus *Orthoflavivirus* within the family *Flaviviridae* and possesses a positive-sense single-stranded RNA genome approximately 11 kilobases in length. The genomic architecture features conserved 5′ and 3′ untranslated regions (UTRs) flanking a single open reading frame (ORF) that encodes a polyprotein precursor. Post-translational processing yields three structural proteins (C, prM, and E) and seven non-structural proteins (NS1, NS2A, NS2B, NS3, NS4A, NS4B, and NS5), which collectively mediate viral replication and pathogenesis [4]. Advances in molecular virology techniques have enabled the comprehensive characterization of JEV evolution. Phylogeographic analyses trace the virus origin to the Malaysia–Philippines archipelago, with global JEV segregating into five distinct genotypes (G1–G5) [5]. Notably, genotype 3 (G3), historically, the predominant genotype across Asia, has been gradually displaced by genotype 1 (G1) since the mid-1990s [6]. Recent phylogenetic studies suggest further subdivision of G1 viruses into two phylogenetically distinct clades: G1a and G1b, exhibiting differential geographic distribution patterns [7].

JEV is a natural focus pathogen and sustains its transmission cycle through mosquito vectors and mammalian hosts within ecosystems [8]. Human and animal infections arise primarily through bites from hematophagous vectors, making the diversity of JEV vectors (e.g., mosquitoes), reservoir hosts, and the spatiotemporal distribution of viral isolates pivotal in shaping its epidemiology. Recent genomic surveillance highlights genotype 1 (G1) replacing genotype 3 (G3) as the predominant circulating lineage, with G1 exhibiting reduced host diversity compared to G3 [9]. However, current understanding remains limited by a critical temporal gap: most data predate 2012, obscuring the ecological dynamics of JEV vectors/hosts and genotype transitions over the past decade. This study analyzes 2012–2022 shifts in JEV transmission through the comparative molecular evolution of full-genome sequences, the spatiotemporal mapping of isolates, and vector–host interactions. A phylogenetic analysis of 2012–2022 genomes confirms sustained divergence between G1 and G3, while the signaling potential emergence of G4 and G5 genotypes in Asia requires vigilance. Mosquitoes—notably *Culex tritaeniorhynchus*—and swine remain central amplification hosts, affirming their indispensable role in viral ecology. Despite JEV’s evolving host adaptations, the mosquito–swine cycle remains its dominant transmission pathway, necessitating sustained vector control and targeted surveillance of these primary transmission routes.

## 2. Materials and Methods

### 2.1. Phylogenetic Analysis of JEV Genome Data Sets from 2012 to 2022

Phylogenetic analyses were performed on a total of 88 full-length genome sequences retrieved from the GenBank database (http://www.ncbi.nlm.nih.gov/, accessed on 1 March 2025). Vaccine or derivative strains, in vitro-cultured isolates, and isolates with sequences containing significant numbers of ambiguous nonstandard nucleotide or amino acid characters were excluded. The genome sequences were isolated from a variety of hosts (mosquitoes, *Culicoides*, *Sus scrofa*, *Anatidae*, *Bos taurus*, *Ovis aries*, *Phocidae*, and humans) with isolation dates ranging from 2012 to 2022. The geographic distribution covered 12 countries/regions: Angola, Australia, Cambodia, China, Chinese Taiwan, India, Indonesia, Japan, Philippines, Singapore, The Republic of Korea, and Thailand. Murray Valley encephalitis virus (MVEV) was used as an outgroup to root in the evolutionary tree. Nucleotide and amino acid sequences were aligned using ClustalW v2.0 and manually refined in MEGA v5.05. Phylogenetic trees were reconstructed for the complete coding regions using the maximum composite likelihood substitution model in MEGA v5.05, with nodal support assessed by 1000 bootstrap replicates. Detailed metadata for all sequences are available upon request (Supplementary Table S1).

### 2.2. Comparative Spatiotemporal Analysis of G1 and G3 JEV Transmission Dynamics: Pre- and Post-2012 Divergence

Viral genome sequences of genotypes 1 (G1), 3 (G3), 4 (G4), and 5 (G5) JEV were retrieved from the GenBank database, encompassing isolates collected between 2012 and 2022 across 13 countries/regions: Angola, Australia, Cambodia, China, Chinese Taiwan, India, Indonesia, Japan, Philippines, Singapore, The Republic of Korea, Thailand, and Vietnam. Sequences lacking critical annotations—such as host/vector associations; collection dates; geographic metadata; or those derived from vaccine strains, laboratory clones, duplicates, or fragmented entries—were systematically excluded. A total of 743 sequences were retained for analysis, including 430 E gene sequences, 88 complete genomes, 65 partial E gene sequences, 19 C-prM sequences, 83 prM sequences, and 58 NS1-NS5 sequences (Supplementary Table S1). To the assess spatiotemporal distribution trends of G1 and G3 isolates before and after 2012, comparative geographic analyses were performed against pre-2012 isolates, referencing historical data from HAN et al. [9]. Phylogenetic and geospatial mapping analyses were conducted using MEGA 11 (for unknown genotype sequence alignment and tree construction) and ArcGIS 10.8 to visualize evolutionary relationships and geographic distribution shifts.

### 2.3. Host and Vector Specificity of G1 and G3 JEV Patterns

To evaluate the host/vector specificity and evolutionary differentiation of Japanese encephalitis virus (JEV) genotype 1 (G1) and genotype 3 (G3) around 2012, based on historical surveillance data from 1930 to 2012 by HAN et al. [9], the G1 and G3 isolates from 743 metadata were classified using PubMed metadata annotations and supplementary literature records to classify their host and media associations. Ecological preferences (e.g., dominant host and primary vector) were compared between 1930–2012 and 2012–2022. Correlations were tabulated to highlight changes in host/carrier tropism between G1 and G3.

## 3. Results

### 3.1. Phylogenetic Analysis of JEV Full-Length Genome from 2012 to 2022

#### 3.1.1. Geographical and Host Distribution of JEV Full-Length Genome from 2012 to 2022

A total of 88 JEV isolates from 2012 to 2022 were obtained from 12 countries and regions, of which China (42, 47.72%) and Japan (14, 15.91%) showed the most, followed by Australia, Chinese Taiwan, Cambodia, and The Republic of Korea (5 each, 5.68%); India (4, 4.55%); Singapore (3, 3.41%); Indonesia (2, 2.27%); and Angola, Philippines, and Thailand (1 each, 1.14%).

A host analysis revealed that 45.45% (40/88) of isolates originated from vector insects, primarily *Culex tritaeniorhynchus* and other *Culex* spp. (34/40, 85.00%), followed by unspecified *Culicidae* (3), *Anopheles sinensis* (2), and *Culicoides* (1). Animal hosts accounted for 42.05% (37/88), predominantly from wild and domestic pigs (*Sus scrofa*: 25; *Sus scrofa domesticus*: 5), with fewer isolates from *Bos taurus* and *Phocidae* (2 each), as well as *Phoca vitulina*, *Ovis aries*, and *Anatidae* (1 each). Human (Homo sapiens) isolates constituted 12.50% (11/88), confirming zoonotic transmission while highlighting the role of mosquito vectors and swine reservoirs in JEV ecology.

#### 3.1.2. Genotypic Diversity and Host/Geographical Associations

**Figure 1:**
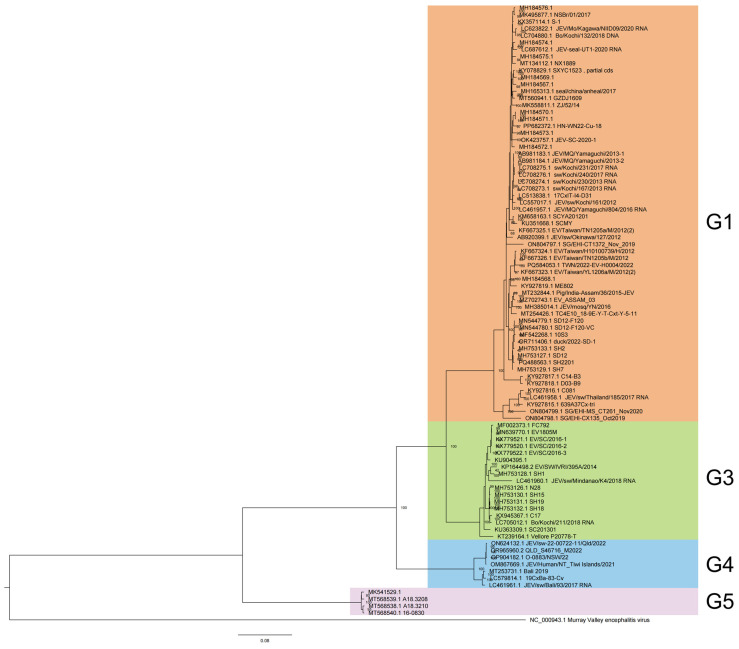
Phylogenetic analysis of JEV genome from 2012 to 2022.

A phylogenetic analysis classified 88 JEV full-length genome sequences from 2012 to 2022 into four genotypes: G1 (60, 68.18%), G3 (17, 19.32%), G4 (7, 7.95%), and G5 (4, 4.55%) (Figure 1), each exhibiting distinct host preferences and geographical clustering.

Among the 60 genotype 1 JEV isolates, vectors represented the highest proportion, at 51.67% (31/60), with *Culex tritaeniorhynchus* being the predominant vector, representing 80.65% (25/31) of vector-associated isolates. The remaining vectors included *Culex* spp. (2), *Culex pipiens pallens* (1), *Culex quinquefasciatus* (1), and an unspecified *Culicidae* species (1). Notably, one full-genome sequence was isolated from *Culicoides* in China in 2017 (GenBank accessions: MH184573), a genus not typically recognized as a primary JEV vector, warranting further investigation into its epidemiological relevance. Animal hosts constituted 40.00% (24/60) of isolates, with swine (*Sus scrofa* and *Sus scrofa domesticus*) dominating this category at 75.00% (18/24). Other animal hosts included *Phocidae* (3), *Bos taurus* (1), *Ovis aries* (1), and *Anatidae* (1). Human cases contributed minimally (8.33%, 5/60), aligning with G1’s zoonotic transmission dynamics, which predominantly involve vectors and animal reservoirs. Among the 17 genotype 3 JEV isolates, animal-derived cases were predominant (58.82%, 10/17), with *Sus scrofa* (swine) serving as the primary reservoir (90.00%, 9/10). The remaining animal host was *Bos taurus* (1). Mosquito vectors accounted for 23.53% (4/17) of isolates, while human cases represented 17.65% (3/17), indicating a slightly higher human involvement compared to G1 but still maintaining a strong zoonotic transmission cycle. In genotype 4, the majority of isolates were obtained from *Sus scrofa* (swine), constituting 42.86% (3/7) of the total. Mosquito vectors and human isolates were equally represented, each contributing 28.57% (2/7). The limited number of G4 isolates suggests a narrower host range or lower detection rates compared to other genotypes. Genotype 5 JEV isolates were primarily associated with mosquito vectors, representing 75.00% (3/4) of cases. Human infections accounted for the remaining 25.00% (1/4). No animal isolates were reported for G5, which may reflect sampling limitations or unique transmission patterns distinct from other genotypes.

G1 60 isolates are the most widespread and were reported from eight countries and regions across the Asia-Pacific region, circulating extensively across East, Southeast, and South Asia. The highest number of isolates came from China (31), followed by Japan (13), Cambodia (5), Chinese Taiwan (5), Singapore (3), The Republic of Korea (1), India (1), and Thailand (1). This widespread isolate distribution highlights G1’s adaptability to diverse ecological settings and its established transmission cycle involving *Culex* mosquitoes and swine reservoirs in endemic regions. G3 17 isolates were detected in five countries, spanning Asia and, notably, Africa. The majority of G3 sequences originated from China (11), with additional isolates from Japan (1), India (3), and the Philippines (1). A significant finding was the first reported JEV detection in Africa, represented by a G3 isolate from Angola [10]. This suggests that G3 shows transcontinental presence, with the first African detection (Angola) raising concerns about JEV’s potential expansion into new regions. G4 and G5 exhibit a more limited distribution, with all seven G4 isolates originating from Southeast Asia and Oceania. The majority were reported from Australia (5), followed by Indonesia (2). All four G5 isolates were obtained from The Republic of Korea. This geographic confinement may reflect limited surveillance, and ecological or host-specific barriers that restrict G4’and G5’s spread compared to more widely distributed genotypes like G1 and G3.

### 3.2. Comparative Spatiotemporal Analysis of G1 and G3 JEV Transmission Dynamics in 2012–2022

**Figure 2:**
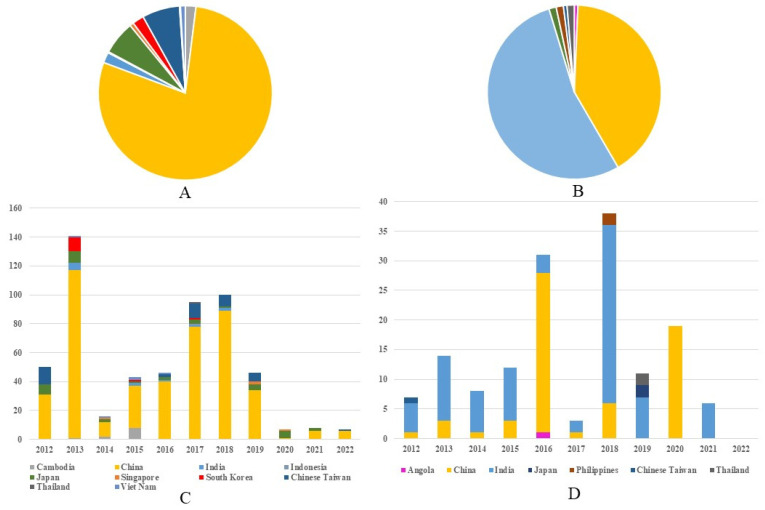
Regional composition of G1 and G3 JE virus isolates in 2012–2022. (**A**) The regional composition ratio of G1 JEV isolates during 2012–2022. (**B**) The regional composition ratio of G3 JEV isolates during 2012–2022. (**C**) The country composition of G1 JEV isolates from each year during 2012–2022. (**D**) The country composition of G3 JEV isolates from each year during 2012 to 2022. Note: The color representations for different countries in (**A**)/(**B**) correspond to those in (**C**)/(**D**), respectively.

Between 2012 and 2022, 559 G1 JEV isolates from 10 countries across Asia were reported. China represented the predominant source, with 440 isolates (78.71%, 440/559), followed distantly by Chinese Taiwan (6.97%, 39/559,) and Japan (6.26%, 35/559). Other reporting countries included The Republic of Korea (12), India (11), Cambodia (11), Vietnam (5), Singapore (4), Indonesia (1), and Thailand (1) (Figure 2A). Continuous annual detection of G1 isolates occurred in both China and Japan (except 2022 in Japan), demonstrating stable endemic circulation in these regions. The temporal distribution revealed two modest peaks in G1 isolation rates—one in 2013 and another spanning 2017–2018—which may correlate with documented adult JE outbreaks in China during these periods [11,12] (Figure 2C). Concurrently, 149 G3 isolates were identified from seven nations spanning Asia and Africa, including Angola’s first reported JEV case. India accounted for the majority of G3 detections (53.69%, 80/149), with China representing the second largest contributor (40.94%, 61/149) (Figure 2B). India maintained near-annual G3 isolation from 2012 to 2021 (with absence only in 2020), while China demonstrated consistent detection through 2018 followed by abrupt cessation (excepting 19 isolates reported in 2020). The temporal pattern for G3 diverged from G1, exhibiting pronounced peaks in 2016 and 2018 that may reflect distinct regional outbreaks or variations in enzootic transmission cycles between the two genotypes. (Figure 2D). Notably, G1 and G3 detection frequencies showed marked decline after 2020, potentially attributable to the COVID-19 pandemic’s impacts on surveillance systems or altered transmission dynamics. These differential epidemiological patterns between G1 and G3 suggest genotype-specific transmission dynamics that warrant further investigation. (Supplementary Tables S2 and S3).

### 3.3. Comparative Analysis of JEV Genotype 1 and 3 Distribution Patterns from 1930 to 2012

**Figure 3:**
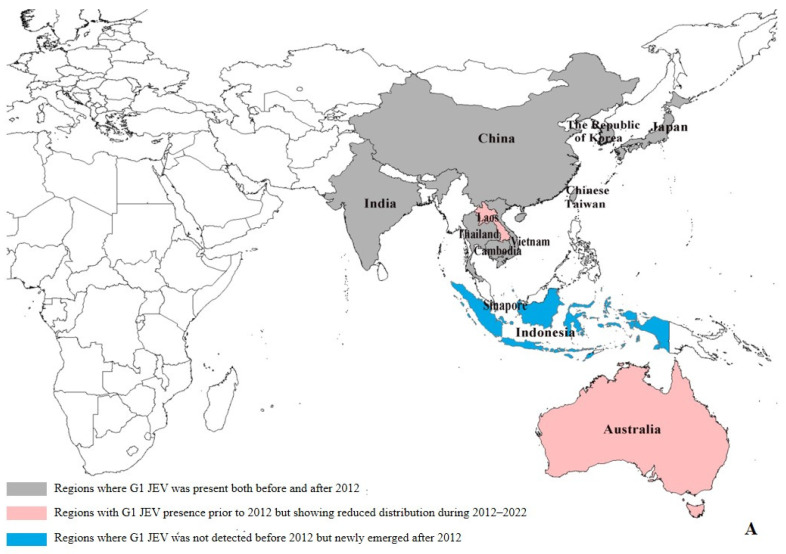

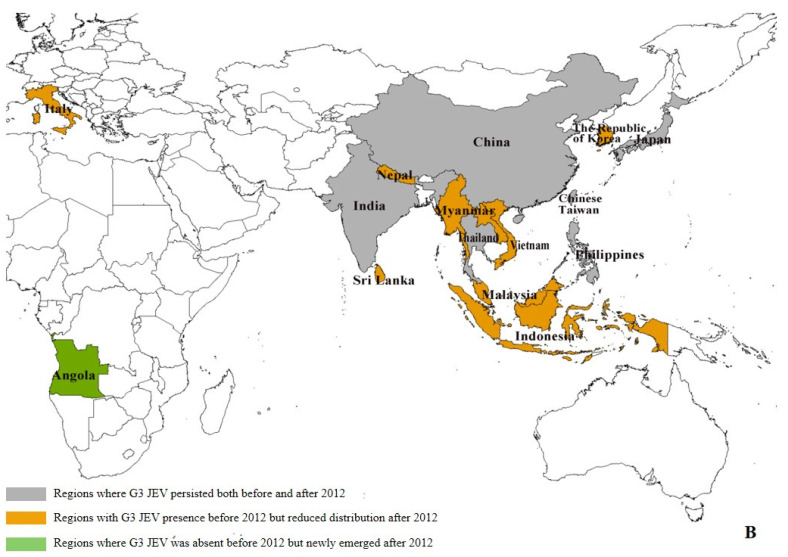
Regional distribution of G1 and G3 JEV from 1930 to 2022 [9]. (**A**) Regional distribution of G1 JEV. Gray areas: regions where G1 JEV was present both before and after 2012; red areas: regions with G1 JEV presence prior to 2012 but showing reduced distribution during 2012–2022; blue areas: regions where G1 JEV was not detected before 2012 but newly emerged after 2012. (**B**) Regional distribution of G3 JEV. Gray areas: regions where G3 JEV persisted both before and after 2012; yellow areas: regions with G3 JEV presence before 2012 but reduced distribution after 2012; green areas: regions where G3 JEV was absent before 2012 but newly emerged after 2012 (e.g., Africa); white areas: Oceania, where G1 and G3 JEV has never been detected.

Our data reveal significant geographical shifts in G1 JEV distribution in the post-2012 period. In East Asia, mainland China, Chinese Taiwan, and Japan maintained their historical G1 predominance, demonstrating persistent endemic circulation. In South Asia, G1 remains potentially present only in India, and even there, at relatively low prevalence levels (pre-2012: 6.39% (29/454), post-2012: 1.97% (11/559)). Notably, we observed emerging G1 hotspots across Southeast Asia, particularly in Singapore (pre-2012:0, post-2012:4) and Indonesia (pre-2012:0, post-2012:1), (Supplementary Table S4), where increased isolation rates may indicate evolving transmission dynamics and potential establishment of new endemic foci (Figure 3A). The post-2012 period shows distinct regional variations in G3 circulation patterns. While mainland China (East Asia) and India (South Asia) maintain active G3 transmission, we documented reduced G3 activity in Japan (pre-2012: 15.03% (66/439), post-2012: 1.34% (2/149)) and Chinese Taiwan (pre-2012: 18.22% (80/439), post-2012: 0.67% (1/149)). In Southeast Asian countries such as Malaysia, Indonesia, Vietnam, Sri Lanka, and Myanmar, as well as in Nepal in South Asia, G3 has not been detected since 2012 (Supplementary Table S4). Our analysis reveals concerning evidence of G3 JEV expansion beyond its traditional endemic range. With the exception of Angola, where the first local human case of G3 has been reported, local zoonotic transmission in Africa has been confirmed [10]. Although G3 JEV was detected in European mosquito populations [13] and bird hosts (GenBank: AF501311–AF501315) prior to 2012, no new evidence of active transmission has been reported in Europe since then. However, these historical detections suggest potential environmental persistence and previously unrecognized enzootic cycles in non-endemic regions. These findings highlight the expanding geographical range of G3 JEV and underscore the need for enhanced global surveillance. (Figure 3B).

### 3.4. Temporal Dynamics of Host-Specific Divergence Between G1 and G3 JEV Isolates

**Figure 4:**
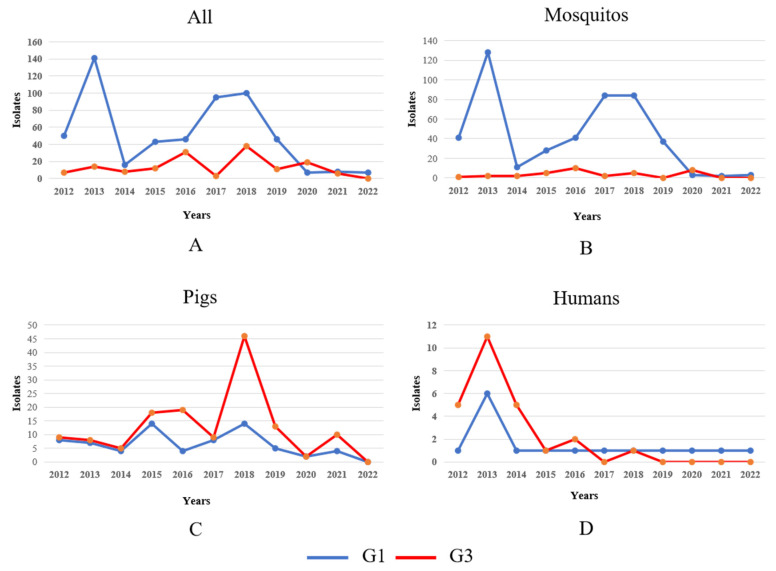
Temporal dynamics of G1 and G3 JEV isolates in different hosts in 2012–2022. Note: The blue and red lines in the figure represent the changes in the number of G1 and G3 JEV isolates, respectively. (**A**) Temporal dynamics of G1 and G3 JEV isolates in mosquitos, pigs, and humans from 2012 to 2022. (**B**) Temporal dynamics of G1 and G3 JEV isolates in mosquito from 2012 to 2022. (**C**) Temporal dynamics of G1 and G3 JEV isolates in pigs from 2012 to 2022. (**D**) Temporal dynamics of G1 and G3 JEV isolates in humans from 2012 to 2022.

Our analysis revealed significant host-specific divergence in JEV isolation patterns between genotypes during the post-2012 surveillance period. While overall isolation rates for both G1 and G3 exhibited interannual fluctuations (Figure 4A), a detailed examination uncovered distinct epidemiological trends across different hosts. Mosquito vectors: G1 isolates from mosquito populations demonstrated a pronounced doubling in detection frequency between 2014 and 2020, suggesting intensified enzootic transmission cycles. In contrast, G3 mosquito isolates persisted at consistently low levels throughout the observation period, indicating limited vector involvement in G3 maintenance (Figure 4B). G3 isolates from swine hosts showed a marked escalation from 2017 to 2020, potentially reflecting emerging porcine amplification dynamics or improved surveillance in key endemic regions (Figure 4C). Both genotypes exhibited a transient surge in human case isolations during 2012–2014, possibly associated with regional outbreak events. This was followed by a substantial and sustained decline in subsequent years, likely attributable to expanded vaccination programs and/or altered zoonotic transmission intensity (Figure 4D). These findings highlight genotype-specific ecological adaptations, with G1 demonstrating increasing vector association while G3 shows stronger porcine host affinity in recent years. The parallel decline in human infections across genotypes post-2014 suggests successful public health interventions may be mitigating spillover risk despite maintained enzootic circulation.

### 3.5. Host Specificity and Genotype Distribution Patterns of G1 and G3 JEV in 1930–2022

#### 3.5.1. Host Specificity of G1 and G3 JEV

From 1930 to 2012, G1 and G3 JEV exhibited distinct host association patterns. A total of 454 G1 isolates were identified from five animal species, while 439 G3 isolates originated from seven host species, indicating broader host diversity for G3 despite fewer total isolates. A detailed analysis revealed genotype-specific reservoir preferences: Mosquito-derived isolates constituted 61% (278/454) of G1 isolates compared to 45% (197/439) for G3. Swine-associated isolates showed marked disparity, with G1 accounting for 26.43% (120/454) and G3 accounting for 12.30% (54/439). Human isolates showed the opposite pattern, with G3 infection accounting for 39.86% (175/439) and G1 infection for 12.30% (54/439). When excluding human-derived isolates (as humans serve as dead-end hosts for JEV), considering only vectors and animal hosts involved in natural transmission and amplification, by 2012, there were 664 isolates, with mosquitoes accounting for 475 isolates (G1: 278, G3: 197), while swine contributed 174 isolates (G1: 120, G3: 54). Other vectors/animals represented 15 isolates. Collectively, mosquitoes and swine comprised 98% (649/664) of all non-human isolates during this period (Table 1).

Our comprehensive surveillance data reveal a significant post-2012 expansion of both G1 and G3 JEV host tropism, with successful establishment in six novel species—the hard tick *Haemaphysalis flava*, domestic cattle (*Bos taurus*), harbor seals (*Phoca vitulina*), pangolins (order *Pholidota*), meerkats (*Suricata suricatta*), and domestic sheep (*Ovis aries*)—increasingly known JEV-competent hosts from 7 to 13 species (71.4% niche expansion). Genotype-specific adaptation patterns emerged: G1 isolates showed preferential association with ixodid ticks and marine mammals (particularly *Phoca vitulina*), while G3 demonstrated specialized tropism for terrestrial species like pangolins and meerkats. While G1 dominates mosquito transmission cycles (82.65%, 462/559) of vector-borne isolates compared to G3’s 23.49% (35/149), the pattern completely reverses in mammalian hosts. G3 shows significantly higher infection rates than G1 in both swine reservoirs (G3: 46.31%, 69/149; G1: 12.52%, 70/559) and human cases (G3: 16.78%, 25/149; G1: 2.86%, 16/559). This represents a dramatic epidemiological shift from the pre-2012 period when G3’s swine infection rate (12.30%) was less than half of G1’s (26.43%). The post-2012 surge in G3’s mammalian infectivity suggests this genotype has undergone evolutionary changes enhancing its adaptation to mammalian hosts, while G1 maintains its traditional dominance in mosquito-mediated transmission cycles. These divergent pathways may reflect distinct evolutionary strategies for viral maintenance and spread between the two genotypes. While the 21 isolates (3.15% of 667 non-human isolates) from novel host species represent a relatively small proportion, they provide compelling evidence of the virus’s evolving ecological plasticity and capacity for cross-species transmission. Importantly, despite these host range expansions, traditional reservoir species maintain their epidemiological predominance, with mosquitoes and swine collectively responsible for 95.35% (636/667) of all non-human isolates. They reveal the remarkable stability of classical transmission cycles even as the virus explores new ecological niches. (Table 1).

#### 3.5.2. Temporal Dynamics of G1 and G3 JEV Transmission Vectors

Prior to 2012, Japanese encephalitis virus (JEV) transmission was mediated by two primary vector groups: mosquitoes (19 identified species) and midges (*Lasiohelea*, *Culicoides* spp.). A genotype-specific analysis revealed distinct vector associations: For G1, 217 mosquito-derived isolates were recorded, with *Culex tritaeniorhynchus* (the principal vector) accounting for 204 isolates, representing 94% (204/217) of all classified G1 mosquito vectors. For G3 JEV, 128 mosquito-derived isolates were identified, of which 95 (74.22%, 95/128) originated from *Cx. tritaeniorhynchus*. Following 2012, JEV transmission vectors diversified to include three arthropod groups—mosquitoes, midges, and ticks—spanning 30 species and yielding 501 isolates. Ticks (Ixodidae family) were newly identified as potential JEV vectors, marking an ecological expansion. For G1 JEV, mosquitoes remained the dominant vectors. When excluding 126 unclassified mosquito specimens, with 333 isolates across 10 species. *Cx. tritaeniorhynchus* continued its prominence, represented 92.19% (307/333) of genetically identified vectors. The secondary vector is *Culex pipiens pallens* (seven), followed by *Culex quinquefasciatus* and *Anopheles sinensis* (four each). For G3 JEV, all 35 isolates were restricted to mosquitoes, distributed across 10 species. *Cx. tritaeniorhynchus* accounted for 7 isolates, while 14 isolates derived from unclassified mosquitoes. The remaining isolates were sporadically detected in species such as *Anopheles sinensis* (three), and *Culex pipiens* and *Culex quinquefasciatus* (two each). Unclassified mosquitoes constituted 27.47% (126/466) of post-2012 G1 isolates, highlighting ongoing challenges in vector species identification. (Table 2).

## 4. Discussion

The genomic surveillance of Japanese encephalitis virus (JEV) from 2012 to 2022 reveals significant epidemiological shifts, with G1 and G3 genotypes maintaining global dominance (accounting for 95.29% of 743 isolates from 13 countries). While G1 persists as endemic in East and South Asia with expanding transmission in Southeast Asia, G3 shows declining prevalence in traditional regions but concerning spread into Africa and Europe, warranting vigilance in historically JEV-free zones. Notably, the re-emergence of rare genotypes marks a pivotal epidemiological transition: G4 was detected during Australia’s 2022 outbreak (with 42 human cases reported across five jurisdictions from January 2021 to June 2022, signaling potential endemic establishment) [14,15], marking its inaugural detection outside historical Asian foci. G5 JEV was initially identified in a fatal 1950s Malaysian case [16]. Since 2010, more than 20 GV isolates have been identified in the Republic of Korea (ROK) [17] and have re-emerged in a 2015 South Korean human infection [18], breaking a 70-year epidemiological silence and demonstrating a genotype shift from GI to GV in the region [19]. Furthermore, these epidemiological trends, coupled with documented G2 circulation in Malaysia, Indonesia, Papua New Guinea, and Australia (1970–2000) [20], raise critical questions about the potential resurgence of G2. Collectively, these findings highlight the evolving public health threat posed by JEV’s genotypic diversification, highlighting the need for continued genomic surveillance and adaptive prevention strategies [21].

Our studies reveal significant evolutionary divergence in host adaptation patterns between G1 and G3 JEV genotypes in the post-2012 era. While G1 maintains strong vector competence, particularly in *Culex tritaeniorhynchus* mosquitoes (accounting for 92.96% of vector-borne isolates), G3 has emerged as the dominant genotype in mammalian hosts, showing significantly higher prevalence in both swine reservoirs (46.31% vs. G1’s 12.52%) and human cases (16.78% vs. 2.86%). This epidemiological shift is corroborated by Chinese serological data (2018–2020) identifying G3 as the predominant human-infecting genotype [22]. However, a significant clinical series documented Guillain–Barré syndrome (GBS) onset following infection with G1 JEV in 2018, revealing an important neurological complication associated with this specific viral genotype [23]. Our study has identified six novel host species for JEV, including ticks (potential arthropod vectors), domestic cattle, harbor seals, pangolins, meerkats, and domestic sheep. Although these new hosts collectively account for only 3.15% of total viral isolates—suggesting their current limited role in natural transmission cycles—their detection represents a significant expansion of JEV host tropism that has emerged after 2012. This finding highlights an important evolutionary development in JEV ecology that warrants continued surveillance. Crucially, conventional mosquito–swine transmission remains the dominant pathway responsible for 95.35% of isolates. Experimental studies confirm that multiple mosquito species can transmit emerging G4 JEV, highlighting the risks of geographic spread [24]. Recent epidemiological studies from China and India demonstrate that JEV exhibits particularly efficient diffusion in regions characterized by warm climates, high pig density, and established populations of competent *Culex* mosquito vectors, with viral spread being further potentiated by both domestic pig trade networks and wild bird migration patterns [25,26]. Collectively, these findings demonstrate that despite JEV’s expanding ecological plasticity through genotype-specific adaptations—with G1 JEV maybe establishing arthropods—marine mammal transmission networks and G3 JEV may develop terrestrial host specialization, and the fundamental maintenance of transmission cycles continues to rely predominantly on the classic Culex–swine pathway, underscoring the critical need for targeted surveillance strategies focusing on these primary vector–host interactions.

The observed surge in G1 isolate detections post-2012 (1930–2012: 454 isolates vs. 2012–2022: 559 isolates) likely reflects the implementation of enhanced nationwide arbovirus surveillance in mainland China between 2012 and 2018. This comprehensive monitoring program successfully identified multiple mosquito–borne viruses, including dengue and West Nile virus, with JEV constituting the majority of viral isolates [27,28]. These surveillance efforts account for mainland China’s substantial contribution of approximately 80% of global JEV isolates during this period. However, temporal disparities in vector–host human case isolation patterns underscore the necessity for integrated longitudinal studies incorporating simultaneous monitoring of mosquito populations, swine reservoirs, and human cases.

While GenBank-derived metadata provide valuable insights into JEV genotypic variations and biogeographical distribution, several limitations must be acknowledged. Variations in national surveillance capacities and uneven geographical/temporal coverage have introduced sampling biases that may distort ecological interpretations. For instance, while Schuh et al.’s E-protein phylogeny proposed distinct G1a/G1b sublineages [29], the absence of complete G1a genomes in our dataset means our whole-genome evolutionary analysis actually characterized the G1b evolutionary cluster. Furthermore, the absence of G2 isolates in current genomic databases necessitates further investigation to determine whether this represents true epidemiological extinction or simply reflects surveillance gaps. Additionally, the global COVID-19 pandemic has significantly compromised post-2020 JEV surveillance data quality and completeness worldwide, resulting in notably scarce genomic sequence availability. This data paucity particularly affects our understanding of recent JEV evolutionary dynamics and genotype distribution patterns. Our country-level mapping approach in Figure 3, while effective for broad visualization, may not completely represent the finer-scale regional variations in JEV distribution patterns. To address the distinct host adaptation patterns of G1 and G3 JEV genotypes, tailored public health measures should include enhanced surveillance of novel hosts (especially marine mammals and ticks) and targeted *Culex* control in G1-affected areas, intensified swine monitoring in G3-endemic regions, while urgently evaluating G3 vaccine efficacy against G1/G2/G4/G5 genotypes to guide multivalent vaccine development—all complementing core Culex–swine transmission control to ensure comprehensive protection against this evolving arboviral threat. These findings emphasize the critical need for strengthened, harmonized surveillance of JEV vectors, animal reservoirs, environmental viral pools, and human cases to enable accurate risk mapping and support evidence-based public health interventions.

## 5. Conclusions

The post-2012 genomic landscape confirms the continued global dominance of G1 and G3 JEV genotypes; G1 maintains strong endemicity in Asia, with emerging South-east Asian hotspots; and G3 demonstrates concerning transcontinental spread to Africa and Europe while documenting the significant re-emergence of previously rare G4 and G5 genotypes, representing a new epidemiological challenge for historically JEV-free regions. This study reveals continued genotype-specific ecological specialization, with G1 maintaining strong mosquito vector associations (particularly *Cx. tritaeniorhynchus*) and G3 predominating in swine reservoirs and human cases, though both genotypes show expanded host ranges through novel arthropod vectors and mammalian species. The mosquito–swine transmission cycle remains the fundamental maintenance mechanism across all genotypes and time periods, demonstrating remarkable ecological stability amid genomic evolution. The convergence of genotypic diversification, vector expansion, and intercontinental transmission underscores the urgent need to establish standardized, integrated “one health” surveillance systems, including vectors, animal hosts, environmental surveillance, and human cases, to accurately track the evolution and transmission dynamics of JEV.

## Figures and Tables

**Table 1:** Differences in host composition for genotype I and III isolates before and after 2012 [9].

Host	G1		G3		G1 & G3	
	1930–2012	2012–2022	1930–2012	2012–2022	1930–2012	2012–2022
Mosquito	278	462	197	35	475	497
Midge	1		2		3	0
Pig	120	70	54	69	174	139
Human	54	16	175	25	229	41
Tick		4				4
Cattle		1		1		2
Seal		3				3
Bat			6	8	6	8
*Pholidota*				7		7
*Suricata suricatta*				2		2
Horse	1		4	1	5	1
Sheep		2		1		3
Bird		1	1		1	1
Total	454	559	439	149	893	708

**Table 2:** Comparison of the range of vector isolates for G1 and G3 JEV before and after 2012 [9].

Subgroup and Species	G1 No. of Isolates (% Isolates)		G3 No. of Isolates (% Isolates)	
	1930–2012	2012–2022	1930–2012	2012–2022
Mosquitos				
*Aedes aegypti*		2 (0.43)		1 (2.86)
*Aedes vexans*			2 (1.56)	
*Aedes lineatopennis*			1 (0.78)	
*Aedes butleri*				
*Anopheles sinensis*	1 (0.46)	4 (0.86)	3 (2.34)	3 (8.57)
*Anopheles minimus*			1 (0.78)	
*Armigeres subalbatus*		1 (0.21)	3 (2.34)	
*Armigeres* spp.	2 (0.92)		4 (3.13)	
*Culex gelidus*	1 (0.46)	1 (0.21)		1 (2.86)
*Culex orientalis*		1 (0.21)		
*Culex pipiens*	1 (0.46)	2 (0.43)		2 (5.71)
*Culex pipiens pallens*	3 (1.38)	7 (1.50)		1 (2.86)
*Culex quinquefasciatus*	1 (0.46)	4 (0.86)	2 (1.56)	2 (5.71)
*Culex tritaeniorhynchus*	204 (93.58)	307 (65.88)	95 (74.22)	7 (20.00)
*Culex vishnui*	1 (0.46)		5 (3.91)	
*Culex bitaeniorhynchus*				
*Culex modestus*	1 (0.46)			
*Culex whitmorei*			1 (0.78)	
*Culex epidesmus*			1 (0.78)	
*Culex fuscocephalus*			1 (0.78)	
*Culex theileri*			3 (2.34)	
*Culex annulus*			2 (1.56)	
*Culex pseudovishnui*	2 (0.92)		2 (1.56)	
*Anopheles vagus*				
*Culex annulirostris*				
*Culex sitiens*				
*Culex* spp.		4 (0.86)		3 (8.57)
*Mansonia uniformis*				1 (2.86)
Unclassified mosquitoe		128 (27.47)		14 (40.00)
Midges				
*Lasiohelea taiwana Shiraki*	1 (0.46)			
Culicoides		1 (0.21)	2 (1.56)	
Ticks				
*Haemaphysalis flava*		4 (0.86)		
Total	218 (100)	466 (100)	128 (100)	35 (100)

## Data Availability

All the data generated during the current study are included in the manuscript and/or the Supplementary data. Additional data related to this article may be requested from the corresponding authors.

## Supplementary Materials

The following supporting information can be downloaded at https://www.mdpi.com/article/10.3390/v17060815/s1; Table S1: Information on the virus isolates analyzed in this study; Table S2: G1 JEV isolates by region from 2012 to 2022; Table S3: G3 JEV isolates by region from 2012 to 2022; Table S4: G1 and G3 historical (1930–2022) virus information.
