# Supplementary material for: Spatiotemporal Distribution and Host–Vector Dynamics of Japanese Encephalitis Virus
Source: Viruses. 2025 Jun 4;17(6):815. doi: 10.3390/v17060815 (PMC12197556; doi:10.3390/v17060815)
Supplement: Supplementary file 1 [file viruses-17-00815-s001.zip › viruses-3595646-supplementary.pdf]

viruses

MDPI

Supplementary Materials

Spatiotemporal Distribution and Host–Vector Dynamics of Japanese Encephalitis Virus

Table S1. Information on the virus isolates analyzed in this study.

No.	Accession	Isolate	Gene	Length	Geo Location	Host	Collection Date	Genotype
1	KT230861	JEV-NIV-149330	C/PrM	367	India	Homo sapiens	2014	3
2	KT230862	JEV-NIV-149325	C/PrM	367	India	Homo sapiens	2014	3
3	KT230863	JEV-NIV-149333	C/PrM	367	India	Homo sapiens	2014	3
4	KT230864	JEV-NIV-149347	C/PrM	367	India	Homo sapiens	2014	3
5	KX000880	JEV02	C/PrM	434	India	Sus scrofa	2015	3
6	KX000881	JEV03	C/PrM	434	India	Equus caballus	2015	3
7	KU748674	JEV01/VPH/MVC/India/2015	C/PrM	427	India	Sus scrofa	2015	3
8	KX119145	JEV04	C/PrM	477	India	Culex	2015	3
9	KX119146	JEV05	C/PrM	477	India	Culex	2015	3
10	KX119147	JEV06	C/PrM	470	India	Culex	2015	3
11	KX119148	JEV07	C/PrM	484	India	Culex quinquefasciatus	2015	3
12	KX119149	JEV08	C/PrM	477	India	Culex quinquefasciatus	2015	3
13	MH193523	YN2017-1	C/PrM	807	China: Yunnan province	Culicidae	2017	1
14	MZ832308	M8982	C/PrM	228	India	Sus scrofa	2021	3
15	MZ832309	R1	C/PrM	218	India	Sus scrofa	2021	3
16	MZ832310	H1	C/PrM	204	India	Sus scrofa	2021	3
17	MZ832311	H2	C/PrM	182	India	Sus scrofa	2021	3
18	MZ832312	H3	C/PrM	253	India	Sus scrofa	2021	3
19	MZ832313	D13	C/PrM	182	India	Sus scrofa	2021	3
20	AB920399	JEV/sw/Okinawa/127/2012	complete	10956	Japan: Okinawa	Sus scrofa	2012	1
21	KF667323	JEV/Chinese Taiwan/YL1206a/M/2012(2)	complete	10914	Chinese Taiwan	Culex tritaeniorhynchus	2012	1
22	KF667324	JEV/Chinese Taiwan/H10100739/H/2012	complete	10914	Chinese Taiwan	Homo sapiens	2012	1
23	KF667325	JEV/Chinese Taiwan/TN1205a/M/2012(2)	complete	10914	Chinese Taiwan	Culex tritaeniorhynchus	2012	1
24	KF667326	JEV/Chinese Taiwan/TN1205b/M/2012	complete	10914	Chinese Taiwan	Culex tritaeniorhynchus	2012	1
25	KM658163	SCYA201201	complete	10965	China	Sus scrofa	2012	1
26	LC557017	JEV/sw/Kochi/161/2012	complete	10221	Japan	Sus scrofa domesticus	2012	1
27	AB981183	JEV/MQ/Yamaguchi/2013-1	complete	10965	Japan: Yamaguchi, Yoshida	Culex tritaeniorhynchus	2013	1
28	AB981184	JEV/MQ/Yamaguchi/2013-2	complete	10965	Japan: Yamaguchi, Yoshida	Culex tritaeniorhynchus	2013	1
29	KY927819	ME802	complete	10864	Cambodia	Homo sapiens	2013	1

30	LC708273	sw/Kochi/167/2013	complete	10749	Japan:Kochi	Sus scrofa domesticus	2013	1
31	LC708274	sw/Kochi/230/2013	complete	10749	Japan:Kochi	Sus scrofa domesticus	2013	1
32	MF542268	10S3	complete	10965	China	Sus scrofa	2013	1
33	KU363309	SC201301	complete	10976	China	Sus scrofa	2013	3
34	KU904395	Japanese encephalitis virus KU904395	complete	10399	China	Culicidae	2013	3
35	KU351668	SCMY	complete	10965	China: Sichuan province	Sus scrofa	2014	1
36	KY927815	639A37Cx-tri	complete	10931	Cambodia	Culex tritaeniorhynchus	2014	1
37	MK558811	ZJ/52/14	complete	10960	China	Culex tritaeniorhynchus	2014	1
38	KP164498	JEV/SW/IVRI/395A/2014	complete	10976	India	Sus scrofa	2014	3
39	KX357114	JS-1	complete	10960	China	Culex tritaeniorhynchus	2015	1
40	KY078829	SXYC1523	complete	10296	China	Culex pipiens pallens	2015	1
41	KY927816	C081	complete	10928	Cambodia	Homo sapiens	2015	1
42	KY927817	C14-B3	complete	10897	Cambodia	Sus scrofa	2015	1
43	KY927818	D03-B9	complete	10896	Cambodia	Sus scrofa	2015	1
44	MH753127	SD12	complete	10965	China	Sus scrofa	2015	1
45	MT232844	Pig/India-Assam/36/2015-JEV	complete	10965	India	Sus scrofa	2015	1
46	MH753126	N28	complete	10961	China	Sus scrofa	2015	3
47	MH753128	SH1	complete	10976	China	Sus scrofa	2015	3
48	MK541529	K15P38	complete	10302	South Korea	Homo sapiens	2015	5
49	MF002373	FC792	complete	10977	China	Sus scrofa	2016	1
50	MH385014	JEV/mosq/YN/2016	complete	10976	China	Culicidae	2016	1
51	MH753129	SH7	complete	10965	China	Culex tritaeniorhynchus	2016	1
52	MH753133	SH2	complete	10965	China	Culex tritaeniorhynchus	2016	1
53	MT560941	GZDJ1609	complete	10912	China	Culex quinquefasciatus	2016	1
54	LC461957	JEV/MQ/Yamaguchi/804/2016	complete	10965	Japan:Yamaguchi, Yoshida	Culex tritaeniorhynchus	2016	1
55	KT239164	Vellore P20778-T	complete	10977	India	Homo sapiens	2016	3
56	KX779520	JEV/SC/2016-2	complete	10714	China: Sichuan	Sus scrofa	2016	3
57	KX779521	JEV/SC/2016-1	complete	10716	China: Sichuan Provence	Sus scrofa	2016	3
58	KX779522	JEV/SC/2016-3	complete	10715	China: Sichuan	Sus scrofa	2016	3
59	KX945367	C17	complete	10961	Angola	Homo sapiens	2016	3
60	MH753130	SH15	complete	10977	China	Anopheles sinensis	2016	3
61	MH753131	SH19	complete	10977	China	Anopheles sinensis	2016	3
62	MH753132	SH18	complete	10977	China	Culex tritaeniorhynchus	2016	3
63	MT568540	16-0830	complete	10963	South Korea: Yongsan	Culex orientalis	2016	5
64	LC513838	17CxIT-I4-D31	complete	10513	Japan: Ishikawa, Monzenmachi	Culex tritaeniorhynchus	2017	1
65	LC708275	sw/Kochi/231/2017	complete	10902	Japan:Kochi	Sus scrofa domesticus	2017	1
66	LC708276	sw/Kochi/240/2017	complete	10817	Japan:Kochi	Sus scrofa domesticus	2017	1
67	MH165313	seal/china/anheal/2017	complete	10965	China	Seal	2017	1
68	MH184567	Japanese encephalitis virus MH184567	complete	10966	China	Culex tritaeniorhynchus	2017	1

69	MH184568	Japanese encephalitis virus MH184568	complete	10965	China	Culex tritaeniorhynchus	2017	1
70	MH184569	Japanese encephalitis virus MH184569	complete	10966	China	Culex tritaeniorhynchus	2017	1
71	MH184570	Japanese encephalitis virus MH184570	complete	10968	China	Culex tritaeniorhynchus	2017	1
72	MH184571	Japanese encephalitis virus MH184571	complete	10967	China	Culex tritaeniorhynchus	2017	1
73	MH184572	Japanese encephalitis virus MH184572	complete	10964	China	Culex tritaeniorhynchus	2017	1
74	MH184573	Japanese encephalitis virus MH184573	complete	10969	China	Culicoides	2017	1
75	MH184574	Japanese encephalitis virus MH184574	complete	10956	China	Culex tritaeniorhynchus	2017	1
76	MH184575	Japanese encephalitis virus MH184575	complete	10964	China	Culex tritaeniorhynchus	2017	1
77	MH184576	Japanese encephalitis virus MH184576	complete	10956	China	Culex tritaeniorhynchus	2017	1
78	MK495877	JNSBr/01/2017	complete	10964	South Korea	Seal	2017	1
79	LC461958	JEV/sw/Thailand/185/2017	complete	10967	Thailand:Nakornnayok province, Banna District	Sus scrofa	2017	1
80	LC461961	JEV/sw/Bali/93/2017 RNA	complete	10970	Indonesia:Bali, Denpasar	Sus scrofa	2017	4
81	LC704880	Bo/Kochi/132/2018 DNA	complete	10822	Japan:Kochi	Bos taurus	2018	1
82	MT134112	NX1889	complete	10967	China: Ningxia	Homo sapiens	2018	1
83	MT254426	TC4E10_18-9E-Y-T-Cxt-Y-5-11	complete	10953	China: Fuyu village, Beihai township, Yunan Province	Culex tritaeniorhynchus	2018	1
84	LC461960	JEV/sw/Mindanao/K4/2018	complete	10976	Philippines:Mindanao, Agusan Del Norte, Butuan City, Kinamlutan	Sus scrofa	2018	3
85	MN639770	JEV1805M	complete	10988	China	Homo sapiens	2018	3
86	MZ702743	JEV_ASSAM_03	complete	10966	India	Sus scrofa	2018	3
87	MT568538	A18.3210	complete	10984	South Korea: Camp Hamphreys	Culex bitaeniorhynchus	2018	5
88	MT568539	A18.3208	complete	10962	South Korea: Camp Hamphreys	Culex bitaeniorhynchus	2018	5
89	MN544779	SD12-F120	complete	10965	China	Sus scrofa	2019	1
90	MN544780	SD12-F120-VC	complete	10965	China	Sus scrofa	2019	1
91	LC705012	Bo/Kochi/211/2018	complete	10897	Japan:Kochi	Bos taurus	2019	3
92	LC579814	19CxBa-83-Cv	complete	10857	Indonesia: Bali, Tabanan	Culex vishnui	2019	4
93	OM867669	21-02409-01	complete	10949	Australia	Homo sapiens	2021	4
94	OP904182	O-0883/NSW/22	complete	10970	Australia	Sus scrofa	2022	4
95	OR965960	QLD_S46716_M2022	complete	10970	Australia	Culicidae	2022	4
96	MT253731	Bali 2019	complete	10970	Australia	Homo sapiens	2019	4
97	LC623822	JEV/Mo/Kagawa/NIID09/2020 RNA	complete	10915	Japan: Kagawa, Sakaide	Culex tritaeniorhynchus	2020	1
98	PQ584053	TWN/2022-EV-H0004/2022	complete	10965	Chinese Taiwan	Homo sapiens	2022	1
99	OK423757	JEV-SC-2020-1	complete	10964	China	Sus scrofa	2020	1
100	LC687612	JEV-seal-UT1-2020	complete	10920	Japan	Phoca vitulina	2021	1
101	ON804798	SG/EHI-CX135_Oct2019	complete	10946	Singapore	Culex	2019	1
102	ON804797	SG/EHI-CT1372_Nov_2019	complete	10949	Singapore	Culex tritaeniorhynchus	2019	1

103	ON804799	SG/EHI-MS_CT261_Nov2020	complete	10941	Singapore	Culex tritaeniorhyn-chus	2020	1
104	PQ488563	SH2201	complete	10893	China	Ovis aries	2022	1
105	PP682372	HN-WN22-Cu-18	complete	10299	China	Culex	2022	1
106	OR711406	duck/2022-SD-1	complete	10965	China	Anatidae	2022	1
107	ON624132	JEV/sw-22-00722-11/Qld/2022	complete	10941	Australia: Queensland	Sus scrofa	2022	4
108	KX118441	LYG-1	E	2250	China: Lianyungang	Culex tritaeniorhyn-chus	2015	1
109	KX118442	LYG-2	E	2253	China: Lianyungang	Culex tritaeniorhyn-chus	2015	1
110	KX118443	LYG-3	E	2241	China: Lianyungang	Culex tritaeniorhyn-chus	2015	1
111	KX118444	LYG-4	E	2253	China: Lianyungang	Culex tritaeniorhyn-chus	2015	1
112	OL700072	XY121909	E	7765	China: Yunnan	Culex pipiens	2018	1
113	MN072896	SaX12-31	E	1500	China	Culex tritaeniorhyn-chus	2012	1
114	MN072897	SaX12-35	E	1500	China	Culex tritaeniorhyn-chus	2012	1
115	MN072898	SaX12-36	E	1500	China	Culex tritaeniorhyn-chus	2012	1
116	MN072899	SaX12-41-2	E	1500	China	Culex tritaeniorhyn-chus	2012	1
117	MN072900	SaX12-44	E	1500	China	Culex tritaeniorhyn-chus	2012	1
118	MN072901	SaX12-119-1	E	1500	China	Anopheles sinensis	2012	1
119	MN072902	SX12-3P-2	E	1500	China	Culex tritaeniorhyn-chus	2012	1
120	MN072903	SX12-3P-8	E	1500	China	Culex tritaeniorhyn-chus	2012	1
121	MN072904	SX12-5P-2	E	1500	China	Culex tritaeniorhyn-chus	2012	1
122	MN072905	SX12-3P-15	E	1500	China	Culex tritaeniorhyn-chus	2012	1
123	MN072906	SX12-5P-29	E	1500	China	Culex tritaeniorhyn-chus	2012	1
124	KP216584	12-YJ016	E	1500	China	Culex tritaeniorhyn-chus	2012	1
125	KP216585	12-YJ017	E	1500	China	Culex tritaeniorhyn-chus	2012	1
126	KP216586	12-YJ021	E	1500	China	Culex tritaeniorhyn-chus	2012	1
127	KP216587	12-YJ022	E	1500	China	Culex tritaeniorhyn-chus	2012	1
128	KP216588	12-YJ023	E	1500	China	Culex pipiens pal-lens	2012	1
129	KP216589	12-YJ030	E	1500	China	Culex tritaeniorhyn-chus	2012	1
130	KP216590	12-YJ033	E	1500	China	Culex tritaeniorhyn-chus	2012	1
131	KP216591	12-YJ038	E	1500	China	Culex pipiens pal-lens	2012	1
132	KP216592	12-YJ044	E	1500	China	Culex tritaeniorhyn-chus	2012	1
133	KP216593	12-YJ054	E	1500	China	Culex tritaeniorhyn-chus	2012	1
134	KP216594	12-YJ075	E	1500	China	Culex tritaeniorhyn-chus	2012	1

135	KP216595	12-YJ078	E	1500	China	Culex tritaeniorhynchus	2012	1
136	KP216596	12-YJ082	E	1500	China	Culex tritaeniorhynchus	2012	1
137	KP216597	12-YJ084	E	1500	China	Culex tritaeniorhynchus	2012	1
138	KP216598	12-LY039	E	1500	China	Culex pipiens pallens	2012	1
139	KJ000029	ZJ12-3	E	1500	China: Zhejiang	Culex tritaeniorhynchus	2012	1
140	KJ000030	ZJ12-4	E	1500	China: Zhejiang	Culex tritaeniorhynchus	2012	1
141	KJ000031	ZJ12-6	E	1500	China: Zhejiang	Culex tritaeniorhynchus	2012	1
142	KJ000032	ZJ12-7	E	1500	China: Zhejiang	Culex pipiens pallens	2012	1
143	KF667301	JEV/Chinese Taiwan/TPC1206a/M/2012	E	1500	Chinese Taiwan	Culex tritaeniorhynchus	2012	1
144	KF667302	JEV/Chinese Taiwan/TPC1207a/M/2012	E	1500	Chinese Taiwan	Culex tritaeniorhynchus	2012	1
145	KF667303	JEV/Chinese Taiwan/YL1206b/M/2012	E	1500	Chinese Taiwan	Culex tritaeniorhynchus	2012	1
146	KF667304	JEV/Chinese Taiwan/TC1206a/M/2012	E	1500	Chinese Taiwan	Culex tritaeniorhynchus	2012	1
147	KF667305	JEV/Chinese Taiwan/TC1206c/M/2012	E	1500	Chinese Taiwan	Culex tritaeniorhynchus	2012	1
148	KF667306	JEV/Chinese Taiwan/TC1206d/M/2012	E	1500	Chinese Taiwan	Culex tritaeniorhynchus	2012	1
149	KF667307	JEV/Chinese Taiwan/TN1205c/M/2012	E	1500	Chinese Taiwan	Culex tritaeniorhynchus	2012	1
150	KF667308	JEV/Chinese Taiwan/HL1205a/M/2012	E	1500	Chinese Taiwan	Culex tritaeniorhynchus	2012	1
151	KF667309	JEV/Chinese Taiwan/TPC1206c/M/2012	E	1500	Chinese Taiwan	Culex tritaeniorhynchus	2012	3
152	KC879324	IND-WB-JE3	E	1500	India	Homo sapiens	2012	3
153	KC879325	IND-WB-JE4	E	1500	India	Homo sapiens	2012	3
154	KC879326	IND-WB-JE5	E	1500	India	Homo sapiens	2012	3
155	KC526871	IND/12/WB/JEV50	E	1500	India: West Bengal, Malda	Homo sapiens	2012	3
156	KC802022	IND/12/WB/JEV51	E	1500	India: West Bengal, Birbhum	Homo sapiens	2012	3
157	KM496505	K12YJ1182	E	1500	South Korea: Yeoju	Culex orientalis	2012	5
158	KJ420589	K12HC959	E	1500	South Korea: Hwacheon	Culex orientalis	2012	5
159	KJ420590	K12AS1148	E	1500	South Korea: Ansan	Culex pipiens	2012	5
160	KJ420591	K12AS1151	E	1500	South Korea: Ansan	Culex orientalis	2012	5
161	KJ420592	K12YJ1203	E	1500	South Korea: Yeoju	Culex orientalis	2012	5
162	KY083680	JEV/LKO/02	E	1500	India	Homo sapiens	2013	1
163	KY083684	JEV/LKO/06	E	1500	India	Homo sapiens	2013	1
164	KY083685	JEV/LKO/07	E	1500	India	Homo sapiens	2013	1
165	KY083686	JEV/LKO/08	E	1500	India	Homo sapiens	2013	1
166	KY083687	JEV/LKO/09	E	1500	India	Homo sapiens	2013	1
167	KM496493	K13BS163	E	1500	South Korea: Busan	Culex tritaeniorhynchus	2013	1
168	KM496494	K13BS168	E	1500	South Korea: Busan	Culex tritaeniorhynchus	2013	1
169	KM496495	K13BS169	E	1500	South Korea: Busan	Culex tritaeniorhynchus	2013	1

170	KM496496	K13BS124	E	1500	South Korea: Busan	Culex tritaeniorhynchus	2013	1
171	KM496497	K13BS132	E	1500	South Korea: Busan	Culex tritaeniorhynchus	2013	1
172	KM496498	K13BS134	E	1500	South Korea: Busan	Culex tritaeniorhynchus	2013	1
173	KM496500	K13JN11	E	1500	South Korea: Hwasun	Culex tritaeniorhynchus	2013	1
174	KM496501	K13GN44	E	1500	South Korea: Haman-gun	Culex tritaeniorhynchus	2013	1
175	KM496502	K13GN46	E	1500	South Korea: Haman-gun	Culex tritaeniorhynchus	2013	1
176	KJ000033	ZJ13-6	E	1500	China: Zhejiang	Culex tritaeniorhynchus	2013	1
177	KJ000034	ZJ13-7	E	1500	China: Zhejiang	Culex tritaeniorhynchus	2013	1
178	KJ000035	ZJ13-9	E	1500	China: Zhejiang	Culex tritaeniorhynchus	2013	1
179	KJ000036	ZJ13-10	E	1500	China: Zhejiang	Culex tritaeniorhynchus	2013	1
180	KJ000037	ZJ13-11	E	1500	China: Zhejiang	Culex tritaeniorhynchus	2013	1
181	KM079080	ZJ13-01	E	1500	China: Zhejiang	Culex tritaeniorhynchus	2013	1
182	KM079081	ZJ13-02	E	1500	China: Zhejiang	Culex tritaeniorhynchus	2013	1
183	KM079082	ZJ13-03	E	1500	China: Zhejiang	Culex tritaeniorhynchus	2013	1
184	KM079083	ZJ13-21	E	1500	China: Zhejiang	Culex tritaeniorhynchus	2013	1
185	KM079084	ZJ13-28	E	1500	China: Zhejiang	Culex tritaeniorhynchus	2013	1
186	KM079085	ZJ13-33	E	1500	China: Zhejiang	Anopheles sinensis	2013	1
187	KM079086	ZJ13-36	E	1500	China: Zhejiang	Culex pipiens pallens	2013	1
188	KJ190833	LiC51/SD/CHN/10	E	1500	China	Culicidae	2013	1
189	KJ190834	LiC68/SD/CHN/10	E	1500	China	Culicidae	2013	1
190	KJ190835	LiC79/SD/CHN/10	E	1500	China	Culicidae	2013	1
191	KJ190836	ZP3/SD/CHN/13	E	1500	China	Culicidae	2013	1
192	KJ190837	KL59/SD/CHN/13	E	1500	China	Culicidae	2013	1
193	KJ190838	KL67/SD/CHN/13	E	1500	China	Culicidae	2013	1
194	KJ190839	KL68/SD/CHN/13	E	1500	China	Culicidae	2013	1
195	KJ190840	KL70/SD/CHN/13	E	1500	China	Culicidae	2013	1
196	KJ190841	KL78/SD/CHN/13	E	1500	China	Culicidae	2013	1
197	KJ190842	KL84/SD/CHN/13	E	1500	China	Culicidae	2013	1
198	KJ190843	KL86/SD/CHN/13	E	1500	China	Culicidae	2013	1
199	KJ190844	KL91/SD/CHN/13	E	1500	China	Culicidae	2013	1
200	KJ190845	KL92/SD/CHN/13	E	1500	China	Culicidae	2013	1
201	KJ190846	KL97/SD/CHN/13	E	1500	China	Culicidae	2013	1
202	KJ190847	KL109/SD/CHN/13	E	1500	China	Culicidae	2013	1
203	KJ190848	KL113/SD/CHN/13	E	1500	China	Culicidae	2013	1
204	KJ190849	KL114/SD/CHN/13	E	1500	China	Culicidae	2013	1
205	KJ190850	KL115/SD/CHN/13	E	1500	China	Culicidae	2013	1
206	KJ190851	KL116/SD/CHN/13	E	1500	China	Culicidae	2013	1
207	KJ190852	RC10/SD/CHN/13	E	1500	China	Culicidae	2013	1
208	KJ190853	RC20/SD/CHN/13	E	1500	China	Culicidae	2013	1
209	KJ190854	RC21/SD/CHN/13	E	1500	China	Culicidae	2013	1
210	KJ190855	RC34/SD/CHN/13	E	1500	China	Culicidae	2013	1
211	KJ190856	RC35/SD/CHN/13	E	1500	China	Culicidae	2013	1

212	KJ190857	RC47/SD/CHN/13	E	1500	China	Culicidae	2013	1
213	KJ190858	RC49/SD/CHN/13	E	1500	China	Culicidae	2013	1
214	KJ190859	RC54/SD/CHN/13	E	1500	China	Culicidae	2013	1
215	KJ190860	RC55/SD/CHN/13	E	1500	China	Culicidae	2013	1
216	KJ190861	VN11/SD/CHN/13	E	1500	China	Culicidae	2013	1
217	KJ190862	VN47/SD/CHN/13	E	1500	China	Culicidae	2013	1
218	KY083679	JEV/LKO/01	E	1500	India	Homo sapiens	2013	3
219	KY083681	JEV/LKO/03	E	1500	India	Homo sapiens	2013	3
220	KY083682	JEV/LKO/04	E	1500	India	Homo sapiens	2013	3
221	KY083683	JEV/LKO/05	E	1500	India	Homo sapiens	2013	3
222	KY083688	JEV/LKO/10	E	1500	India	Homo sapiens	2013	3
223	KY083689	JEV/LKO/11	E	1500	India	Homo sapiens	2013	3
224	KY083690	JEV/LKO/12	E	1500	India	Homo sapiens	2013	3
225	KY083691	JEV/LKO/13	E	1500	India	Homo sapiens	2013	3
226	KY083692	JEV/LKO/14	E	1500	India	Homo sapiens	2013	3
227	KY083693	JEV/LKO/15	E	1500	India	Homo sapiens	2013	3
228	KY083694	JEV/LKO/16	E	1500	India	Homo sapiens	2013	3
229	KM897135	hunanhuaihua	E	1500	China	Culex tritaeniorhynchus	2013	3
230	KM496503	K13GB57	E	1500	South Korea: Gyeongsan-si	Culex tritaeniorhynchus	2013	5
231	MN072907	HuN14-44	E	1500	China	Culex quinquefasciatus	2014	1
232	LC079039	JEV/sw/Okinawa/186/2014	E	1500	Japan:Okinawa	Sus scrofa	2014	1
233	KP876007	2014.1694	E	1500	Viet Nam: South	Homo sapiens	2014	1
234	KM576774	ZJ14-06	E	1500	China: Zhejiang	Culex tritaeniorhynchus	2014	1
235	KM576775	ZJ14-07	E	1500	China: Zhejiang	Culex tritaeniorhynchus	2014	1
236	KM576776	ZJ14-09	E	1500	China: Zhejiang	Culex tritaeniorhynchus	2014	1
237	KM576777	ZJ14-10	E	1500	China: Zhejiang	Culex tritaeniorhynchus	2014	1
238	KM576778	ZJ14-52	E	1500	China: Zhejiang	Culex tritaeniorhynchus	2014	1
239	KM576779	ZJ14-56	E	1500	China: Zhejiang	Culex tritaeniorhynchus	2014	1
240	KX275306	46B/2014	E	1491	India	Mansonia uniformis	2014	3
241	MT219999	Pig/India-Assam/39/2015-JEV	E	1581	India	Sus scrofa	2015	1
242	MK095778	ZJ-YW-48-15	E	1500	China: Zhejiang	Culex tritaeniorhynchus	2015	1
243	MK095779	ZJ-XJ-31-15	E	1500	China: Zhejiang	Culex tritaeniorhynchus	2015	1
244	MK095780	ZJ-YW-16-15	E	1500	China: Zhejiang	Culex tritaeniorhynchus	2015	1
245	MK095781	ZJ-YW-14-15	E	1500	China: Zhejiang	Culex tritaeniorhynchus	2015	1
246	MK095782	ZJ-YW-11-15	E	1500	China: Zhejiang	Culex tritaeniorhynchus	2015	1
247	MK095783	ZJ-XJ-5-15	E	1500	China: Zhejiang	Culex tritaeniorhynchus	2015	1
248	MK095784	ZJ-XJ-4-15	E	1500	China: Zhejiang	Culex tritaeniorhynchus	2015	1
249	MK095785	ZJ-XJ-3-15	E	1500	China: Zhejiang	Culex tritaeniorhynchus	2015	1
250	MK095786	ZJ-XJ-2-16	E	1500	China: Zhejiang	Culex tritaeniorhynchus	2015	1

251	MK095787	ZJ-XJ-1-17	E	1500	China: Zhejiang	Culex tritaeniorhynchus	2015	1
252	MK095788	ZJ-YW-54-15	E	1500	China: Zhejiang	Culex tritaeniorhynchus	2015	1
253	MK095789	ZJ-YW-36-15	E	1500	China: Zhejiang	Culex tritaeniorhynchus	2015	1
254	MK095790	ZJ-XJ-35-15	E	1500	China: Zhejiang	Culex tritaeniorhynchus	2015	1
255	MK095791	ZJ-XJ-32-15	E	1500	China: Zhejiang	Culex tritaeniorhynchus	2015	1
256	MK095792	ZJ-XJ-30-15	E	1500	China: Zhejiang	Culex tritaeniorhynchus	2015	1
257	MK095793	ZJ-XJ-28-15	E	1500	China: Zhejiang	Culex tritaeniorhynchus	2015	1
258	MK095794	ZJ-XJ-23-15	E	1500	China: Zhejiang	Culex tritaeniorhynchus	2015	1
259	KY927860	HNML2	E	1500	China	Culicidae	2015	1
260	KY927861	HNML3	E	1500	China	Sus scrofa	2015	1
261	KY927814	D08-B9	E	1500	Cambodia	Sus scrofa	2015	1
262	KY078827	SXYC1546	E	1500	China	Culex	2015	1
263	KY078828	SXYC1548	E	1500	China	Culex	2015	1
264	KX774636	HNML1	E	1500	China	Culicidae	2015	1
265	LC075515	JEV/sw/Okinawa/153/2015	E	1500	Japan:Okinawa	Sus scrofa	2015	1
266	KX275302	170/2015	E	1504	India	Homo sapiens	2015	3
267	MZ733971	TC2016-1/Chinese Taiwan/2016/Sw	E	1500	Chinese Taiwan	Sus scrofa	2016	1
268	MZ733972	TC2016-2/Chinese Taiwan/2016/Sw	E	1500	Chinese Taiwan	Sus scrofa	2016	1
269	MT220000	Pig/India-Assam/87/2016-JEV	E	1581	India	Sus scrofa	2016	1
270	MG673536	QP5E_16-7L-Q-Cup-R-4-1	E	1500	China: Qingpu Dist., Shanghai	Culex pipiens pallens	2016	1
271	MG673537	HP4A_16-7-H-Cut-C-5-2	E	1500	China: Huangpu Dist., Shanghai	Culex tritaeniorhynchus	2016	1
272	MK095855	ZJ-YW-1-16	E	1500	China: Zhejiang	Culex tritaeniorhynchus	2016	1
273	MK095856	ZJ-YW-4-16	E	1500	China: Zhejiang	Culex tritaeniorhynchus	2016	1
274	MK095857	ZJ-YW-7-16	E	1500	China: Zhejiang	Culex tritaeniorhynchus	2016	1
275	MK095858	ZJ-YW-8-16	E	1500	China: Zhejiang	Culex tritaeniorhynchus	2016	1
276	MK095859	ZJ-YW-9-16	E	1500	China: Zhejiang	Culex tritaeniorhynchus	2016	1
277	MK095860	ZJ-YW-12-16	E	1500	China: Zhejiang	Culex tritaeniorhynchus	2016	1
278	MK095861	ZJ-YW-16-16	E	1500	China: Zhejiang	Culex tritaeniorhynchus	2016	1
279	MK095862	ZJ-YW-17-16	E	1500	China: Zhejiang	Culex tritaeniorhynchus	2016	1
280	MK095863	ZJ-YW-19-16	E	1500	China: Zhejiang	Culex tritaeniorhynchus	2016	1
281	MK095864	ZJ-YW-21-16	E	1500	China: Zhejiang	Culex tritaeniorhynchus	2016	1
282	MK095865	ZJ-YW-32-16	E	1500	China: Zhejiang	Culex tritaeniorhynchus	2016	1
283	MK095866	ZJ-YW-34-16	E	1500	China: Zhejiang	Culex tritaeniorhynchus	2016	1

284	MK095867	ZJ-YW-44-16	E	1500	China: Zhejiang	Culex tritaeniorhynchus	2016	1
285	MK095868	ZJ-YW-66-16	E	1500	China: Zhejiang	Culex tritaeniorhynchus	2016	1
286	MK095869	ZJ-YW-75-16	E	1500	China: Zhejiang	Culex tritaeniorhynchus	2016	1
287	MK095870	ZJ-YW-82-16	E	1500	China: Zhejiang	Culex tritaeniorhynchus	2016	1
288	MK095871	ZJ-JD-14-16	E	1500	China: Zhejiang	Culex tritaeniorhynchus	2016	1
289	MK095872	ZJ-JD-16-16	E	1500	China: Zhejiang	Culex tritaeniorhynchus	2016	1
290	MK095873	ZJ-JD-22-16	E	1500	China: Zhejiang	Culex tritaeniorhynchus	2016	1
291	MK095874	ZJ-JD-29-16	E	1500	China: Zhejiang	Culex tritaeniorhynchus	2016	1
292	MK095875	ZJ-JD-54-16	E	1500	China: Zhejiang	Culex tritaeniorhynchus	2016	1
293	MK095876	ZJ-JD-55-16	E	1500	China: Zhejiang	Culex tritaeniorhynchus	2016	1
294	MF979778	GZDJ1608	E	1500	China: Guizhou	Culex quinquefasciatus	2016	1
295	MF979780	GZDJ1648	E	1500	China: Guizhou	Culex quinquefasciatus	2016	1
296	LC461956	JEV/MQ/Yamaguchi/803/2016	E	1500	Japan:Yamaguchi, Yoshida	Culex tritaeniorhynchus	2016	1
297	MG673538	SJ11A_16-8-S-Cup-C-1-3	E	1500	China: Songjiang Dist., Shanghai	Culex pipiens pallens	2016	3
298	MG673539	SJ11B_16_8-S-Cut-C-1-1	E	1500	China: Songjiang Dist., Shanghai	Culex tritaeniorhynchus	2016	3
299	KY807144	FCG792	E	1500	China: Guangxi	Sus scrofa	2016	3
300	MZ733961	TC2017-1/Chinese Taiwan/2017/Sw	E	1500	Chinese Taiwan	Sus scrofa	2017	1
301	MZ733962	TC2017-2/Chinese Taiwan/2017/Sw	E	1500	Chinese Taiwan	Sus scrofa	2017	1
302	MZ733963	TC2017-3/Chinese Taiwan/2017/Sw	E	1500	Chinese Taiwan	Sus scrofa	2017	1
303	MZ733964	TC2017-4/Chinese Taiwan/2017/Sw	E	1500	Chinese Taiwan	Sus scrofa	2017	1
304	MZ733965	TC2017-1/Chinese Taiwan/2017/Mq	E	1500	Chinese Taiwan	Culicidae	2017	1
305	MZ733966	TC2017-2/Chinese Taiwan/2017/Mq	E	1500	Chinese Taiwan	Culicidae	2017	1
306	MZ733967	TC2017-3/Chinese Taiwan/2017/Mq	E	1500	Chinese Taiwan	Culicidae	2017	1
307	MZ733968	TC2017-4/Chinese Taiwan/2017/Mq	E	1500	Chinese Taiwan	Culicidae	2017	1
308	MZ733969	TC2017-5/Chinese Taiwan/2017/Mq	E	1500	Chinese Taiwan	Culicidae	2017	1
309	MZ733970	TC2017-6/Chinese Taiwan/2017/Mq	E	1500	Chinese Taiwan	Culicidae	2017	1
310	MT220001	Pig/India-Assam/96/2017-JEV	E	1581	India	Sus scrofa	2017	1
311	MK095795	ZJ-YW-287-17	E	1500	China: Zhejiang	Culex tritaeniorhynchus	2017	1
312	MK095796	ZJ-CX-274-17	E	1500	China: Zhejiang	Culex tritaeniorhynchus	2017	1
313	MK095797	ZJ-CX-271-17	E	1500	China: Zhejiang	Culex tritaeniorhynchus	2017	1

314	MK095798	ZJ-CX-266-17	E	1500	China: Zhejiang	Culex tritaeniorhynchus	2017	1
315	MK095799	ZJ-JD-179-17	E	1500	China: Zhejiang	Culex tritaeniorhynchus	2017	1
316	MK095800	ZJ-JD-178-17	E	1500	China: Zhejiang	Culex tritaeniorhynchus	2017	1
317	MK095801	ZJ-JD-175-17	E	1500	China: Zhejiang	Culex tritaeniorhynchus	2017	1
318	MK095802	ZJ-JD-172-17	E	1500	China: Zhejiang	Culex tritaeniorhynchus	2017	1
319	MK095803	ZJ-JD-163-17	E	1500	China: Zhejiang	Culex tritaeniorhynchus	2017	1
320	MK095804	ZJ-JD-161-17	E	1500	China: Zhejiang	Culex tritaeniorhynchus	2017	1
321	MK095805	ZJ-JD-160-17	E	1500	China: Zhejiang	Culex tritaeniorhynchus	2017	1
322	MK095806	ZJ-JD-159-17	E	1500	China: Zhejiang	Culex tritaeniorhynchus	2017	1
323	MK095807	ZJ-CX-141-17	E	1500	China: Zhejiang	Culex tritaeniorhynchus	2017	1
324	MK095808	ZJ-CX-138-17	E	1500	China: Zhejiang	Culex tritaeniorhynchus	2017	1
325	MK095809	ZJ-CX-128-17	E	1500	China: Zhejiang	Culex tritaeniorhynchus	2017	1
326	MK095810	ZJ-CX-119-17	E	1500	China: Zhejiang	Culex tritaeniorhynchus	2017	1
327	MK095811	ZJ-JD-108-17	E	1500	China: Zhejiang	Culex tritaeniorhynchus	2017	1
328	MK095812	ZJ-JD-96-17	E	1500	China: Zhejiang	Culex tritaeniorhynchus	2017	1
329	MK095813	ZJ-JD-94-17	E	1500	China: Zhejiang	Culex tritaeniorhynchus	2017	1
330	MK095814	ZJ-JD-88-17	E	1500	China: Zhejiang	Culex tritaeniorhynchus	2017	1
331	MK095815	ZJ-JD-82-17	E	1500	China: Zhejiang	Culex tritaeniorhynchus	2017	1
332	MK095816	ZJ-YW-62-17	E	1500	China: Zhejiang	Culex tritaeniorhynchus	2017	1
333	MK095817	ZJ-CX-60-17	E	1500	China: Zhejiang	Culex tritaeniorhynchus	2017	1
334	MK095818	ZJ-CX-56-17	E	1500	China: Zhejiang	Culex tritaeniorhynchus	2017	1
335	MK095819	ZJ-CX-53-17	E	1500	China: Zhejiang	Culex tritaeniorhynchus	2017	1
336	MK095820	ZJ-CX-52-17	E	1500	China: Zhejiang	Culex tritaeniorhynchus	2017	1
337	MK095821	ZJ-CX-51-17	E	1500	China: Zhejiang	Culex tritaeniorhynchus	2017	1
338	MK095822	ZJ-CX-50-17	E	1500	China: Zhejiang	Culex tritaeniorhynchus	2017	1
339	MK095823	ZJ-CX-49-17	E	1500	China: Zhejiang	Culex tritaeniorhynchus	2017	1
340	MK095824	ZJ-CX-46-17	E	1500	China: Zhejiang	Culex tritaeniorhynchus	2017	1
341	MK095825	ZJ-CX-41-17	E	1500	China: Zhejiang	Culex tritaeniorhynchus	2017	1
342	MK095826	ZJ-JD-28-17	E	1500	China: Zhejiang	Culex tritaeniorhynchus	2017	1

343	MK095827	ZJ-JD-24-17	E	1500	China: Zhejiang	Culex tritaeniorhyn-chus	2017	1
344	MK095828	ZJ-JD-23-17	E	1500	China: Zhejiang	Culex tritaeniorhyn-chus	2017	1
345	MK095829	ZJ-YW-16-17	E	1500	China: Zhejiang	Culex tritaeniorhyn-chus	2017	1
346	MK095830	ZJ-YW-10-17	E	1500	China: Zhejiang	Culex tritaeniorhyn-chus	2017	1
347	MK095831	ZJ-YW-2-17	E	1500	China: Zhejiang	Culex tritaeniorhyn-chus	2017	1
348	MK095832	ZJ-YW-389-17	E	1500	China: Zhejiang	Culex tritaeniorhyn-chus	2017	1
349	MK095833	ZJ-YW-384-17	E	1500	China: Zhejiang	Culex tritaeniorhyn-chus	2017	1
350	MK095834	ZJ-YW-368-17	E	1500	China: Zhejiang	Culex tritaeniorhyn-chus	2017	1
351	MK095835	ZJ-YW-357-17	E	1500	China: Zhejiang	Culex tritaeniorhyn-chus	2017	1
352	MK095836	ZJ-YW-348-17	E	1500	China: Zhejiang	Culex tritaeniorhyn-chus	2017	1
353	MK095837	ZJ-YW-347-17	E	1500	China: Zhejiang	Culex tritaeniorhyn-chus	2017	1
354	MK095838	ZJ-YW-327-17	E	1500	China: Zhejiang	Culex tritaeniorhyn-chus	2017	1
355	MK095839	ZJ-YW-326-17	E	1500	China: Zhejiang	Culex tritaeniorhyn-chus	2017	1
356	MK095840	ZJ-YW-325-17	E	1500	China: Zhejiang	Culex tritaeniorhyn-chus	2017	1
357	MK095841	ZJ-YW-324-17	E	1500	China: Zhejiang	Culex tritaeniorhyn-chus	2017	1
358	MK095842	ZJ-YW-319-17	E	1500	China: Zhejiang	Culex tritaeniorhyn-chus	2017	1
359	MK095843	ZJ-YW-316-17	E	1500	China: Zhejiang	Culex tritaeniorhyn-chus	2017	1
360	MK095844	ZJ-YW-303-17	E	1500	China: Zhejiang	Culex tritaeniorhyn-chus	2017	1
361	MK095845	ZJ-YW-297-17	E	1500	China: Zhejiang	Culex tritaeniorhyn-chus	2017	1
362	MK095846	ZJ-YW-295-17	E	1500	China: Zhejiang	Culex tritaeniorhyn-chus	2017	1
363	MK095847	ZJ-YW-291-17	E	1500	China: Zhejiang	Culex tritaeniorhyn-chus	2017	1
364	MK095848	ZJ-YW-290-17	E	1500	China: Zhejiang	Culex tritaeniorhyn-chus	2017	1
365	MK095849	ZJ-YW-309-17	E	1500	China: Zhejiang	Culex tritaeniorhyn-chus	2017	1
366	MK095850	ZJ-YW-307-17	E	1500	China: Zhejiang	Culex tritaeniorhyn-chus	2017	1
367	MK095851	ZJ-YW-306-17	E	1500	China: Zhejiang	Culex tritaeniorhyn-chus	2017	1
368	MK095852	ZJ-YW-305-17	E	1500	China: Zhejiang	Culex tritaeniorhyn-chus	2017	1
369	MK095853	ZJ-YW-315-17	E	1500	China: Zhejiang	Culex tritaeniorhyn-chus	2017	1
370	MK095854	ZJ-YW-313-17	E	1500	China: Zhejiang	Culex tritaeniorhyn-chus	2017	1
371	LC461962	JEV/sw/Bali/94/2017	E	1500	Indonesia:Bali, Denpasar	Sus scrofa	2017	4
372	MT075621	TC2018PigOS-1	E	1500	Chinese Taiwan	Suidae	2018	1

373	MT075622	TC2018PigOS-2	E	1500	Chinese Taiwan	Suidae	2018	1
374	MT075623	TC2018PigOS-3	E	1500	Chinese Taiwan	Suidae	2018	1
375	MT075624	TC2018-1	E	1500	Chinese Taiwan	Culex tritaeniorhyn-chus	2018	1
376	MT075625	TC2018-2	E	1500	Chinese Taiwan	Culex tritaeniorhyn-chus	2018	1
377	MT075626	TC2018-3	E	1500	Chinese Taiwan	Culex tritaeniorhyn-chus	2018	1
378	MT075627	TC2018-4	E	1500	Chinese Taiwan	Culex tritaeniorhyn-chus	2018	1
379	MT075628	TC2018-5	E	1500	Chinese Taiwan	Culex tritaeniorhyn-chus	2018	1
380	MW246758	JS,QH_D3_18-8E-JS-Cxt-C-8-4	E	1498	China: Jinshan District, Shanghai Municipality	Culex tritaeniorhyn-chus	2018	1
381	MW246759	HB_B4_18-7E-HZ-C-Y-5-4	E	1498	China: Zaoyang county, Hubei Province	Culex tritaeniorhyn-chus	2018	1
382	MT254462	TC2G5_18-8E-Y-T-Cxt-Y-5-19	E	1468	China: Fuyu village, Beihai township, Yunan Province	Culex tritaeniorhyn-chus	2018	1
383	MT254463	TC2F6_18-8E-Y-T-Cxt-Y-5-3	E	1468	China: Fuyu village, Beihai township, Yunan Province	Culex tritaeniorhyn-chus	2018	1
384	MT254464	TC2H11_18-8E-Y-T-Cxt-Y-5-44	E	1468	China: Fuyu village, Beihai township, Yunan Province	Culex tritaeniorhyn-chus	2018	1
385	MT254465	TC2B4_18-8M-Y-T-Cxt-Y-5-10	E	1468	China: Fuyu village, Beihai township, Yunan Province	Culex tritaeniorhyn-chus	2018	1
386	MT254466	TC1H10_18-8M-Y-T-Cxt-Y-5-11	E	1468	China: Fuyu village, Beihai township, Yunan Province	Culex tritaeniorhyn-chus	2018	1
387	MT254468	TC4C11_18-9E-Y-T-Cxt-Y-5-12	E	1468	China: Fuyu village, Beihai township, Yunan Province	Culex tritaeniorhyn-chus	2018	1
388	MT254469	TC4D5_18-9E-Y-T-Cxt-Y-5-28	E	1468	China: Fuyu village, Beihai township, Yunan Province	Culex tritaeniorhyn-chus	2018	1
389	MT254470	TC4A5_18-9L-Y-T-Cxt-Y-5-1	E	1468	China: Fuyu village, Beihai township, Yunan Province	Culex tritaeniorhyn-chus	2018	1
390	MN752873	HNLH01	E	1500	China: Henan, Luohe	Sus scrofa	2018	1
391	MN752874	HNLH02	E	1500	China: Henan, Luohe	Sus scrofa	2018	1
392	MN752875	HNXX	E	1500	China: Henan, Xinxiang	Sus scrofa	2018	1
393	MN752876	HNXY	E	1500	China: Henan, Xinyang	Sus scrofa	2018	1
394	MN752877	LNDL1	E	1500	China: Liaoning, Dalian	Culicidae	2018	1
395	MN752878	GXGG	E	1500	China: Guangxi, Gui-gang	Sus scrofa	2018	1
396	MN752879	NMGBT	E	1500	China: Neimenggu, Baotou	Culicidae	2018	1
397	MN752880	GXQZ	E	1500	China: Guangxi, Qingzhou	Sus scrofa	2018	1
398	MN752881	GXNN	E	1500	China: Guangxi, Nan-ning	Sus scrofa	2018	1
399	MN752882	LNDL2	E	1500	China: Liaoning, Dalian	Culicidae	2018	1
400	MT681200	HNLH1809	E	1500	China: Henan, Luohe	Sus scrofa	2018	1
401	MT681201	HNLH1805	E	1500	China: Henan, Luohe	Sus scrofa	2018	1
402	MK095877	ZJ-JY-16-18	E	1500	China: Zhejiang	Culex tritaeniorhyn-chus	2018	1

403	MK095878	ZJ-JY-22-18	E	1500	China: Zhejiang	Culex tritaeniorhyn-chus	2018	1
404	MK095879	ZJ-JY-26-18	E	1500	China: Zhejiang	Culex tritaeniorhyn-chus	2018	1
405	MK095880	ZJ-JY-28-18	E	1500	China: Zhejiang	Culex tritaeniorhyn-chus	2018	1
406	MK095881	ZJ-JY-30-18	E	1500	China: Zhejiang	Culex tritaeniorhyn-chus	2018	1
407	MK095882	ZJ-JY-40-18	E	1500	China: Zhejiang	Culex tritaeniorhyn-chus	2018	1
408	MK095883	ZJ-JY-44-18	E	1500	China: Zhejiang	Culex tritaeniorhyn-chus	2018	1
409	MK095884	ZJ-JY-51-18	E	1500	China: Zhejiang	Culex tritaeniorhyn-chus	2018	1
410	MK095885	ZJ-JY-52-18	E	1500	China: Zhejiang	Culex tritaeniorhyn-chus	2018	1
411	MK095886	ZJ-JY-53-18	E	1500	China: Zhejiang	Culex tritaeniorhyn-chus	2018	1
412	MK095887	ZJ-JY-66-18	E	1500	China: Zhejiang	Culex tritaeniorhyn-chus	2018	1
413	MK095888	ZJ-JY-75-18	E	1500	China: Zhejiang	Culex tritaeniorhyn-chus	2018	1
414	MK095889	ZJ-JY-76-18	E	1500	China: Zhejiang	Culex tritaeniorhyn-chus	2018	1
415	MK095890	ZJ-JY-85-18	E	1500	China: Zhejiang	Culex tritaeniorhyn-chus	2018	1
416	MK095891	ZJ-JY-98-18	E	1500	China: Zhejiang	Culex tritaeniorhyn-chus	2018	1
417	MK095892	ZJ-JY-99-18	E	1500	China: Zhejiang	Culex tritaeniorhyn-chus	2018	1
418	MK095893	ZJ-JY-100-18	E	1500	China: Zhejiang	Culex tritaeniorhyn-chus	2018	1
419	MK095894	ZJ-JY-115-18	E	1500	China: Zhejiang	Culex tritaeniorhyn-chus	2018	1
420	MK095895	ZJ-JY-116-18	E	1500	China: Zhejiang	Culex tritaeniorhyn-chus	2018	1
421	MK095896	ZJ-JY-130-18	E	1500	China: Zhejiang	Culex tritaeniorhyn-chus	2018	1
422	MK095897	ZJ-JY-134-18	E	1500	China: Zhejiang	Culex tritaeniorhyn-chus	2018	1
423	MK095898	ZJ-JY-143-18	E	1500	China: Zhejiang	Culex tritaeniorhyn-chus	2018	1
424	MK095899	ZJ-JY-149-18	E	1500	China: Zhejiang	Culex tritaeniorhyn-chus	2018	1
425	MK095900	ZJ-JY-151-18	E	1500	China: Zhejiang	Culex tritaeniorhyn-chus	2018	1
426	MK095901	ZJ-JY-161-18	E	1500	China: Zhejiang	Culex tritaeniorhyn-chus	2018	1
427	MK095902	ZJ-JY-166-18	E	1500	China: Zhejiang	Culex tritaeniorhyn-chus	2018	1
428	MK095903	ZJ-JY-192-18	E	1500	China: Zhejiang	Culex tritaeniorhyn-chus	2018	1
429	MK095904	ZJ-JY-220-18	E	1500	China: Zhejiang	Culex tritaeniorhyn-chus	2018	1
430	MK095905	ZJ-JY-234-18	E	1500	China: Zhejiang	Culex tritaeniorhyn-chus	2018	1
431	MK095906	ZJ-JY-245-18	E	1500	China: Zhejiang	Culex tritaeniorhyn-chus	2018	1

432	MK095907	ZJ-JY-246-18	E	1500	China: Zhejiang	Culex tritaeniorhynchus	2018	1
433	MK095908	ZJ-JY-254-18	E	1500	China: Zhejiang	Culex tritaeniorhynchus	2018	1
434	MK095909	ZJ-JY-255-18	E	1500	China: Zhejiang	Culex tritaeniorhynchus	2018	1
435	MK095910	ZJ-CX-81-18	E	1500	China: Zhejiang	Culex tritaeniorhynchus	2018	1
436	MK095911	ZJ-CX-83-18	E	1500	China: Zhejiang	Culex tritaeniorhynchus	2018	1
437	MK095912	ZJ-WY-315-18	E	1500	China: Zhejiang	Culex tritaeniorhynchus	2018	1
438	MK095913	ZJ-WY-325-18	E	1500	China: Zhejiang	Culex tritaeniorhynchus	2018	1
439	MK095914	ZJ-WY-326-18	E	1500	China: Zhejiang	Culex tritaeniorhynchus	2018	1
440	MK095915	ZJ-YW-432-18	E	1500	China: Zhejiang	Culex tritaeniorhynchus	2018	1
441	MK095916	ZJ-YW-437-18	E	1500	China: Zhejiang	Culex tritaeniorhynchus	2018	1
442	LC461959	JEV/sw/Mindanao/K3/2018	E	1500	Philippines:Mindanao, Agusan Del Norte, Butuan City, Kinamlutan	Sus scrofa	2018	3
443	MT075618	TC2019PigOS-1	E	1500	Chinese Taiwan	Suidae	2019	1
444	MT075619	TC2019PigOS-2	E	1500	Chinese Taiwan	Suidae	2019	1
445	MT075620	TC2019PigOS-3	E	1500	Chinese Taiwan	Suidae	2019	1
446	MT075629	TC2019-1	E	1500	Chinese Taiwan	Culex tritaeniorhynchus	2019	1
447	MT075630	TC2019-2	E	1500	Chinese Taiwan	Culex tritaeniorhynchus	2019	1
448	MT075631	TC2019-3	E	1500	Chinese Taiwan	Culex tritaeniorhynchus	2019	1
449	LC632497	IM-OI27	E	1500	Japan: Ehime, Imabari	Haemaphysalis flava	2019	1
450	LC632498	IM-OI34	E	1500	Japan: Ehime, Imabari	Haemaphysalis flava	2019	1
451	LC632499	IM-OI35	E	1500	Japan: Ehime, Imabari	Haemaphysalis flava	2019	1
452	LC533131	JEV/Hu/Hiroshima/NIID78/2019	E	1500	Japan	Homo sapiens	2019	1
453	MN403060	ZJ/YQ/21/19	E	1500	China: Zhejiang	Culex tritaeniorhynchus	2019	1
454	MN403061	ZJ/YQ/187/1	E	1500	China: Zhejiang	Culex tritaeniorhynchus	2019	1
455	MN403062	ZJ/YQ/190/19	E	1500	China: Zhejiang	Culex tritaeniorhynchus	2019	1
456	MN403063	ZJ/JY/18/19	E	1500	China: Zhejiang	Culex tritaeniorhynchus	2019	1
457	MN403064	ZJ/JY/22/19	E	1500	China: Zhejiang	Culex tritaeniorhynchus	2019	1
458	MN403065	ZJ/JY/24/19	E	1500	China: Zhejiang	Culex tritaeniorhynchus	2019	1
459	MN403066	ZJ/JY/25/19	E	1500	China: Zhejiang	Culex tritaeniorhynchus	2019	1
460	MN403067	ZJ/JY/32/19	E	1500	China: Zhejiang	Culex tritaeniorhynchus	2019	1
461	MN403068	ZJ/JY/33/19	E	1500	China: Zhejiang	Culex tritaeniorhynchus	2019	1

462	MN403069	ZJ/JY/39/19	E	1500	China: Zhejiang	Culex tritaeniorhyn-chus	2019	1
463	MN403070	ZJ/JY/42/19	E	1500	China: Zhejiang	Culex tritaeniorhyn-chus	2019	1
464	MN403071	ZJ/JY/43/19	E	1500	China: Zhejiang	Culex tritaeniorhyn-chus	2019	1
465	MN403072	ZJ/JY/47/19	E	1500	China: Zhejiang	Culex tritaeniorhyn-chus	2019	1
466	MN403073	ZJ/JY/50/19	E	1500	China: Zhejiang	Culex tritaeniorhyn-chus	2019	1
467	MN403074	ZJ/JY/55/19	E	1500	China: Zhejiang	Culex tritaeniorhyn-chus	2019	1
468	MN403075	ZJ/JY/59/19	E	1500	China: Zhejiang	Culex tritaeniorhyn-chus	2019	1
469	MN403076	ZJ/JY/63/19	E	1500	China: Zhejiang	Culex tritaeniorhyn-chus	2019	1
470	MN403077	ZJ/JY/64/19	E	1500	China: Zhejiang	Culex tritaeniorhyn-chus	2019	1
471	MN403078	ZJ/JY/70/19	E	1500	China: Zhejiang	Culex tritaeniorhyn-chus	2019	1
472	MN403079	ZJ/JY/74/19	E	1500	China: Zhejiang	Culex tritaeniorhyn-chus	2019	1
473	MN403080	ZJ/JY/75/19	E	1500	China: Zhejiang	Culex tritaeniorhyn-chus	2019	1
474	MN403081	ZJ/JY/78/19	E	1500	China: Zhejiang	Culex tritaeniorhyn-chus	2019	1
475	MN403082	ZJ/JY/80/19	E	1500	China: Zhejiang	Culex tritaeniorhyn-chus	2019	1
476	MN403083	ZJ/JY/82/19	E	1500	China: Zhejiang	Culex tritaeniorhyn-chus	2019	1
477	MN403084	ZJ/JY/86/19	E	1500	China: Zhejiang	Culex tritaeniorhyn-chus	2019	1
478	MN403085	ZJ/JY/91/19	E	1500	China: Zhejiang	Culex tritaeniorhyn-chus	2019	1
479	MN403086	ZJ/JY/94/19	E	1500	China: Zhejiang	Culex tritaeniorhyn-chus	2019	1
480	MN403087	ZJ/JY/100/19	E	1500	China: Zhejiang	Culex tritaeniorhyn-chus	2019	1
481	MN403088	ZJ/JY/104/19	E	1500	China: Zhejiang	Culex tritaeniorhyn-chus	2019	1
482	MN403089	ZJ/JY/112/19	E	1500	China: Zhejiang	Culex tritaeniorhyn-chus	2019	1
483	MN403090	ZJ/JY/122/19	E	1500	China: Zhejiang	Culex tritaeniorhyn-chus	2019	1
484	MN403091	ZJ/JY/123/19	E	1500	China: Zhejiang	Culex tritaeniorhyn-chus	2019	1
485	LC571943	sw/Kochi/492/2018	E	1500	Japan:Kochi	Sus scrofa domesti-cus	2019	3
486	LC632500	IM-OI106 E	E	1500	Japan: Ehime, Imabari	Haemaphysalis flava	2020	1
487	LC623659	JEV/Hu/Oka-yama/NIID13/2020	E	1500	Japan: Okayama	Homo sapiens	2020	1
488	OM416151	JEV-China/P2020E-3	E	1500	China	Pholidota	2020	3
489	OM416152	JEV-China/P2020E-2	E	1500	China	Pholidota	2020	3
490	OM416153	JEV-China/P2020E-1	E	1500	China	Pholidota	2020	3
491	OM799545	JEV-China/CT2016E-1	E	1500	China	Culicidae	2020	3
492	OM799546	JEV-China/CT2016E-2	E	1500	China	Culicidae	2020	3
493	OM799547	JEV-China/CT2016E-3	E	1500	China	Culicidae	2020	3
494	OM799548	JEV-China/B2016E-1	E	1500	China	Chiroptera	2020	3

495	OM799549	JEV-China/B2016E-2	E	1500	China	Chiroptera	2020	3
496	OM799550	JEV-China/B2016E-3	E	1500	China	Chiroptera	2020	3
497	OM799551	JEV-China/B2016E-4	E	1500	China	Chiroptera	2020	3
498	OM799552	JEV-China/W2016E-1	E	1500	China	Culicidae	2020	3
499	OM799553	JEV-China/W2016E-2	E	1500	China	Culicidae	2020	3
500	OQ472009	NJ-1	E	1500	China	Culex tritaeniorhyn-chus	2022	1
501	OQ472010	SQ-1	E	1500	China	Culex tritaeniorhyn-chus	2022	1
502	ON568658	HuN-LD-Mosquito-2021	E	1500	China	Culicidae	2021	1
503	ON568659	HuN-YY-Pig-2021	E	1500	China	Sus scrofa	2021	1
504	ON568660	HuN-CS-Pig-2021	E	1500	China	Sus scrofa	2021	1
505	ON568661	HuN-HH-Mosquito-2021	E	1500	China	Culicidae	2021	1
506	ON568662	HuN-ZZ-Pig-2021	E	1500	China	Sus scrofa	2021	1
507	ON568663	HuN-HH-Pig-2021	E	1500	China	Sus scrofa	2021	1
508	PQ488580	SH2202	E	1449	China	Ovis aries	2022	1
509	MZ868506	Sangju-v1	E	1855	South Korea	Culex orientalis	2020	5
510	MZ868507	Sangju-v2	E	1855	South Korea	Culex orientalis	2020	5
511	MW030461	JEV-D2	E	8067	China: Yunnan prov-ince	Anopheles sinensis	2018	1
512	MN529589	XJ1	E	1990	China	Culex pipiens	2018	3
513	MH193526	YN2017-4	E	2001	China: Yunnan prov-ince	Culicidae	2017	1
514	MH193527	YN2017-5	E	2001	China: Yunnan prov-ince	Culicidae	2017	1
515	MH193528	YN2017-6	E	2001	China: Yunnan prov-ince	Culicidae	2017	1
516	MH193529	YN2017-7	E	2001	China: Yunnan prov-ince	Culicidae	2017	1
517	LC095857	JaNP95-12	E	1500	Japan:Nagasaki, Isahaya	Sus scrofa	2012	1
518	LC095858	JaNP105-12	E	1500	Japan:Nagasaki, Isahaya	Sus scrofa	2012	1
519	LC095859	JaNP110-12	E	1500	Japan:Nagasaki, Isahaya	Sus scrofa	2012	1
520	LC095860	JaNP114-12	E	1500	Japan:Nagasaki, Isahaya	Sus scrofa	2012	1
521	LC095861	JaNP119-12	E	1500	Japan:Nagasaki, Isahaya	Sus scrofa	2012	1
522	LC095862	JaNP90-13	E	1500	Japan:Nagasaki, Isahaya	Sus scrofa	2013	1
523	LC095863	JaNP71G-13	E	1500	Japan:Nagasaki, Goto	Sus scrofa	2013	1
524	LC095864	JaNAr10G-13	E	1500	Japan:Nagasaki, Goto	Sus scrofa	2013	1
525	LC095865	JaNP82G-14	E	1500	Japan:Nagasaki, Goto	Sus scrofa	2014	1
526	KT346354	SG/EHI-CT1710-Jun 2014	E	1500	Singapore	Culex tritaeniorhyn-chus	2014	1
527	MF526897	K14CB53	E	1500	South Korea: Chung-cheongbuk-do	Culex pipiens	2014	5
528	MF526898	K14CB803	E	1500	South Korea: Chung-cheongbuk-do	Culex pipiens	2014	5
529	MF526899	K14JB241	E	1500	South Korea: Jeollabuk-do	Culex pipiens	2014	5
530	MF526902	K15JN03	E	1500	South Korea: Jeollanam-do	Culex pipiens	2015	1
531	MF526900	K15GN163	E	1500	South Korea: Gyeong-sangnam-do	Culex pipiens	2015	5

532	MF526901	K15JB132	E	1500	South Korea: Jeollabuk-do	Culex tritaeniorhynchus	2015	5
533	MG644382	Yunnan2016-1	E	1500	China	Culicidae	2016	1
534	MG644383	Yunnan2016-2	E	1500	China	Culicidae	2016	1
535	MG644384	Yunnan2016-3	E	1500	China	Culicidae	2016	1
536	MG644385	Yunnan2016-4	E	1500	China	Culicidae	2016	1
537	MG644386	Yunnan2016-5	E	1500	China	Culicidae	2016	1
538	KF992844	JEV-GZ	E（partial）	421	China	swine	2012	3
539	KJ420593	K12YJ1174	E（partial）	495	South Korea: Yeoju	Culex orientalis	2012	5
540	MG637052	05VZ-75-29-L13	E（partial）	297	Viet Nam	Sus scrofa	2013	1
541	KM496499	K13GW46	E（partial）	456	South Korea: Hoengseong	Culex orientalis	2013	1
542	KM496504	K13GB63	E（partial）	556	South Korea: Gyeongsan-si	Culex tritaeniorhynchus	2013	5
543	KU507208	JD-6	E（partial）	455	China	Culex tritaeniorhynchus	2014	1
544	KU168745	JD-15	E（partial）	1455	China	Culex tritaeniorhynchus	2014	3
545	KJ947880	TNJECH1	E（partial）	282	India	Homo sapiens	2014	3
546	KT946905	HBLY2015	E（partial）	210	China	Ovis aries	2015	3
547	KY807134	792	E（partial）	453	China: Guangxi	Sus scrofa	2016	3
548	KY807135	867-1F1	E（partial）	453	China: Guangxi	Sus scrofa	2016	3
549	KY807136	867-2A	E（partial）	453	China: Guangxi	Sus scrofa	2016	3
550	KY807137	874C-C	E（partial）	453	China: Guangxi	Sus scrofa	2016	3
551	KY807138	890	E（partial）	453	China: Guangxi	Sus scrofa	2016	3
552	KY807139	904A	E（partial）	453	China: Guangxi	Sus scrofa	2016	3
553	KY807140	904B-C	E（partial）	453	China: Guangxi	Sus scrofa	2016	3
554	KY807141	904C-A	E（partial）	453	China: Guangxi	Sus scrofa	2016	3
555	KY807142	905B	E（partial）	453	China: Guangxi	Sus scrofa	2016	3
556	KY807143	905D-C	E（partial）	453	China: Guangxi	Sus scrofa	2016	3
557	KY611855	MPB2	E（partial）	489	India	Sus scrofa	2016	3
558	KY419514	GS01	E（partial）	490	India	Culex gelidus	2016	3
559	MK032889	107/Jambi/2017/Mosquito	E（partial）	601	Indonesia	Culex gelidus	2017	1
560	MW793348	West Bengal	E（partial）	573	India	Culex tritaeniorhynchus	2017	3
561	MK491507	JEV/SW/MKGT73/2017	E（partial）	392	India: Odisha	Sus scrofa	2017	3
562	MH193525	YN2017-3	E（partial）	1064	China: Yunnan province	Culicidae	2017	3
563	MK421340	JEV/SW/MKG524/2018	E（partial）	392	India: Odisha	Sus scrofa	2018	1
564	MH376692	ABTCVKVJE001	E（partial）	390	India	Sus scrofa	2018	1
565	MW246757	JS,QH_B4_18-7E-JS-Cxt-C-9-6	E（partial）	787	China: Jinshan District, Shanghai Municipality	Culex tritaeniorhynchus	2018	1
566	MK571539	JEV/SW/MB2/2018	E（partial）	304	India: Manipur	Sus scrofa	2018	3
567	MK952775	JEV/SW/MB4/2018	E（partial）	379	India: Manipur	Sus scrofa	2018	3
568	MK952776	JEV/SW/MB3/2018	E（partial）	363	India: Manipur	Sus scrofa	2018	3
569	MK962309	JEV/SW/MB5/2018	E（partial）	392	India: Manipur	Sus scrofa	2018	3
570	MK962310	JEV/SW/MB6/2018	E（partial）	391	India: Manipur	Sus scrofa	2018	3
571	MK975821	JEV/SW/MB7/2018	E（partial）	363	India: Manipur	Sus scrofa	2018	3
572	MK975822	JEV/SW/MB8/2018	E（partial）	364	India: Manipur	Sus scrofa	2018	3
573	MK975823	JEV/SW/MB9/2018	E（partial）	363	India: Manipur	Sus scrofa	2018	3
574	MK975824	JEV/SW/MB10/2018	E（partial）	363	India: Manipur	Sus scrofa	2018	3
575	MN010526	JEV/SW/ImphalT-3/2018	E（partial）	375	India: Manipur	Sus scrofa	2018	3
576	MN010527	JEV/SW/ImphalT-41/2018	E（partial）	391	India: Manipur	Sus scrofa	2018	3
577	MN010528	JEV/SW/ImphalT-25/2018	E（partial）	387	India: Manipur	Sus scrofa	2018	3

578	MN010529	JEV/SW/ImphalT-59/2018	E（partial）	391	India: Manipur	Sus scrofa	2018	3
579	MN029013	JEV/SW/ImphalT-4/2018	E（partial）	391	India: Manipur	Sus scrofa	2018	3
580	MN029014	JEV/SW/ImphalT-5/2018	E（partial）	392	India: Manipur	Sus scrofa	2018	3
581	MN029015	JEV/SW/ImphalT-60/2018	E（partial）	392	India: Manipur	Sus scrofa	2018	3
582	MN029016	JEV/SW/ImphalT-61/2018	E（partial）	392	India: Manipur	Sus scrofa	2018	3
583	MN029017	JEV/SW/ImphalT-62/2018	E（partial）	391	India: Manipur	Sus scrofa	2018	3
584	MN029018	JEV/SW/ImphalT-64/2018	E（partial）	393	India: Manipur	Sus scrofa	2018	3
585	MN029019	JEV/SW/IW28/2018	E（partial）	392	India: Manipur	Sus scrofa	2018	3
586	MK518053	JEV/SW/MB1/2018	E（partial）	391	India:Manipur	Sus scrofa	2018	3
587	MK682387	ABTCVKVJE002	E（partial）	390	India	Sus scrofa	2018	3
588	MK692888	ABTCVKVJE003	E（partial）	390	India	Sus scrofa	2018	3
589	MK692889	ABTCVKVJE004	E（partial）	390	India	Sus scrofa	2018	3
590	MK692890	ABTCVKVJE005	E（partial）	390	India	Sus scrofa	2018	3
591	MK692891	ABTCVKVJE006	E（partial）	390	India	Sus scrofa	2018	3
592	MK692892	ABTCVKVJE007	E（partial）	390	India	Sus scrofa	2018	3
593	MK692893	ABTCVKVJE008	E（partial）	390	India	Sus scrofa	2018	3
594	MK692894	ABTCVKVJE009	E（partial）	390	India	Sus scrofa	2018	3
595	MW201807	AJE2	E（partial）	241	India	Sus scrofa	2019	3
596	MW201808	AJE3	E（partial）	244	India	Sus scrofa	2019	3
597	MK940903	JEV/SW/MKG475/2019	E（partial）	392	India: Odisha	Sus scrofa	2019	3
598	MK940904	JEV/SW/MKG478/2019	E（partial）	391	India: Odisha	Sus scrofa	2019	3
599	MK940905	JEV/SW/MKG480/2019	E（partial）	392	India: Odisha	Sus scrofa	2019	3
600	MN115386	JEV/SW/MKG519/2019	E（partial）	391	India: Odisha	Sus scrofa	2019	3
601	MN115387	JEV/SW/MKG524/2019	E（partial）	387	India: Odisha	Sus scrofa	2019	3
602	LC716349	JEV/Hu/Naga-saki/NIID09/2021	E（partial）	359	Japan: Nagasaki	Homo sapiens	2021	1
603	OM799554	JEV-China/CT2016NS1-1	NS1	1056	China	Culicidae	2016	3
604	OM799555	JEV-China/CT2016NS1-2	NS1	1056	China	Culicidae	2016	3
605	OM799556	JEV-China/CT2016NS1-3	NS1	1056	China	Culicidae	2016	3
606	OM799557	JEV-China/CT2016NS1-4	NS1	1056	China	Culicidae	2016	3
607	OM799558	JEV-China/B2016NS1-1	NS1	1056	China	Chiroptera	2016	3
608	OM799559	JEV-China/B2016NS1-2	NS1	1056	China	Chiroptera	2016	3
609	OM799560	JEV-China/B2016NS1-3	NS1	1056	China	Chiroptera	2016	3
610	OM799561	JEV-China/B2016NS1-4	NS1	1056	China	Chiroptera	2016	3
611	OM799562	JEV-China/W2016NS2a-1	NS2a	681	China	Culicidae	2020	3
612	OM799563	JEV-China/W2016NS2a-2	NS2a	681	China	Culicidae	2020	3
613	OM799564	JEV-China/W2016NS2a-3	NS2a	681	China	Culicidae	2020	3
614	MW030468	JEV-G3/YN	NS2A, NS2B, and NS3	1612	China: Yunnan province	Anopheles sinensis	2018	3

615	MW030462	JEV-C3	NS2B and NS3	1100	China: Yunnan province	Aedes aegypti	2018	1
616	KY927809	B14-B04	NS3	161	Cambodia	Sus scrofa	2014	1
617	KY927810	C12-B01	NS3	161	Cambodia	Sus scrofa	2015	1
618	KY927811	D08-B09	NS3	161	Cambodia	Sus scrofa	2015	1
619	KY927812	D15-B12	NS3	162	Cambodia	Sus scrofa	2015	1
620	KY927813	C04-B06	NS3	158	Cambodia	Sus scrofa	2015	1
621	OM416147	JEV-China/P2020NS4a-4	NS4a	378	China	Pholidota	2020	3
622	OM416148	JEV-China/P2020NS4a-3	NS4a	378	China	Pholidota	2020	3
623	OM416149	JEV-China/P2020NS4a-2	NS4a	378	China	Pholidota	2020	3
624	OM416150	JEV-China/P2020NS4a-1	NS4a	378	China	Pholidota	2020	3
625	KY099618	Japanese encephalitis virus KY099618	NS5	1723	Viet Nam: Mekong Delta	Homo sapiens	2016	1
626	MG686629	PD3G_16-9-S-Cut-R-10-3	NS5	221	China: Songjiang Dist., Shanghai	Culex tritaeniorhynchus	2016	1
627	MG686630	PD3H_16-9-S-Cut-C-4-1	NS5	221	China: Songjiang Dist., Shanghai	Culex tritaeniorhynchus	2016	1
628	MG686631	PD8F_16-9E-P-Cut-C-2-21	NS5	221	China: Pudong Dist., Shanghai	Culex tritaeniorhynchus	2016	1
629	MF594404	HK-JEV01	NS5	167	Hong Kong	Homo sapiens	2017	1
630	MT254429	TC2F12_18-8E-Y-T-Cxt-Y-5-30	NS5	264	China: Fuyu village, Beihai township, Yunnan Province	Culex tritaeniorhynchus	2018	1
631	MT254430	TC2F6_18-8E-Y-T-Cxt-Y-5-35	NS5	264	China: Fuyu village, Beihai township, Yunnan Province	Culex tritaeniorhynchus	2018	1
632	MT254432	TC2A1_18-8M-Y-T-Cxt-Y-5-8	NS5	264	China: Fuyu village, Beihai township, Yunnan Province	Culex tritaeniorhynchus	2018	1
633	MT254435	TC2B10_18-8M-Y-T-Cxt-Y-5-21	NS5	264	China: Fuyu village, Beihai township, Yunnan Province	Culex tritaeniorhynchus	2018	1
634	MT254436	TC1H8_18-8M-Y-T-Cxt-Y-5-25	NS5	264	China: Fuyu village, Beihai township, Yunnan Province	Culex tritaeniorhynchus	2018	1
635	MT254437	TC4B3_18-9E-Y-T-Cxt-Y-5-7	NS5	264	China: Fuyu village, Beihai township, Yunnan Province	Culex tritaeniorhynchus	2018	1
636	MT254440	TC4E7_18-9E-Y-T-Cxt-Y-5-21	NS5	264	China: Fuyu village, Beihai township, Yunnan Province	Culex tritaeniorhynchus	2018	1
637	MW030469	JEV-F1/YN	NS5	869	China: Yunnan province	Culex tritaeniorhynchus	2018	1
638	MN379741	MH32	NS5	265	China: Yunnan	Culex tritaeniorhynchus	2018	1
639	MN379742	MH110	NS5	265	China: Yunnan	Culex tritaeniorhynchus	2018	1
640	MN379743	MH119	NS5	265	China: Yunnan	Culex tritaeniorhynchus	2018	1
641	MN379744	MH127	NS5	265	China: Yunnan	Anopheles sinensis	2018	1
642	MN379745	MH135	NS5	265	China: Yunnan	Culex tritaeniorhynchus	2018	1
643	MN379746	MH136	NS5	263	China: Yunnan	Culex tritaeniorhynchus	2018	1

644	MN379747	MH143	NS5	265	China: Yunnan	Culex tritaeniorhynchus	2018	1
645	MN379748	MH153	NS5	256	China: Yunnan	Culex tritaeniorhynchus	2018	1
646	MN534364	XJ2	NS5	791	China	Culex pipiens	2018	3
647	MZ027345	CP01 Meerkat-THA-2019	NS5	191	Thailand	Suricata suricatta	2019	3
648	MZ027346	CP02 Meerkat-THA-2019	NS5	191	Thailand	Suricata suricatta	2019	3
649	MZ868499	Sangju-1	NS5	264	South Korea	Culex orientalis	2020	5
650	MZ868500	Sangju-2	NS5	264	South Korea	Culex orientalis	2020	5
651	MZ868501	Sangju-3	NS5	264	South Korea	Culex orientalis	2020	5
652	MZ868502	Sangju-4	NS5	264	South Korea	Culex orientalis	2020	5
653	MZ868503	Sangju-5	NS5	264	South Korea	Culex orientalis	2020	5
654	MZ868504	Sangju-6	NS5	264	South Korea	Culex orientalis	2020	5
655	MZ868505	Sangju-7	NS5	264	South Korea	Culex orientalis	2020	5
656	MW030493	JEV-G1/YN	NS5、NS2A, NS2B, NS3, NS4B	6167	China: Yunnan province	Aedes aegypti	2018	3
657	MW030490	JEV-C1/YN	NS5、NS2B, NS3	5954	China: Yunnan province	Aedes aegypti	2018	1
658	MW030492	JEV-E1/YN	NS5、NS2B, NS3	6057	China: Yunnan province	Culex tritaeniorhynchus	2018	1
659	MW030494	JEV-G2/YN	NS5、NS2B, NS3	5089	China: Yunnan province	Armigeres subalbatus	2018	1
660	MW030491	JEV-C2/YN	NS5、NS2B, NS3, NS4B	5857	China: Yunnan province	Culex tritaeniorhynchus	2018	3
661	KJ190866	ZP3/SD/CHN/10	PrM	650	China	Culicidae	2013	1
662	KJ190867	rc6/SD/CHN/10	PrM	650	China	Culicidae	2013	1
663	KJ190868	rc7/SD/CHN/10	PrM	650	China	Culicidae	2013	1
664	KJ190869	rc10/SD/CHN/10	PrM	650	China	Culicidae	2013	1
665	KJ190870	rc12/SD/CHN/10	PrM	650	China	Culicidae	2013	1
666	KJ190871	KL5/SD/CHN/10	PrM	650	China	Culicidae	2013	1
667	KJ190872	KL6/SD/CHN/10	PrM	650	China	Culicidae	2013	1
668	KJ190873	KL7/SD/CHN/10	PrM	650	China	Culicidae	2013	1
669	KJ190874	KL25/SD/CHN/10	PrM	650	China	Culicidae	2013	1
670	KJ190875	KL33/SD/CHN/10	PrM	650	China	Culicidae	2013	1
671	KJ190876	KL37/SD/CHN/10	PrM	650	China	Culicidae	2013	1
672	KJ190877	KL46/SD/CHN/10	PrM	650	China	Culicidae	2013	1
673	KJ190878	KL4/SD/CHN/10	PrM	650	China	Culicidae	2013	1
674	KJ190879	KL8/SD/CHN/10	PrM	650	China	Culicidae	2013	1
675	KJ190880	KL10/SD/CHN/10	PrM	650	China	Culicidae	2013	1
676	KJ190881	KL12/SD/CHN/10	PrM	650	China	Culicidae	2013	1
677	KJ190882	KL13/SD/CHN/10	PrM	650	China	Culicidae	2013	1
678	KJ190883	KL14/SD/CHN/10	PrM	650	China	Culicidae	2013	1
679	KJ190884	KL15/SD/CHN/10	PrM	650	China	Culicidae	2013	1
680	KJ190885	KL16/SD/CHN/10	PrM	650	China	Culicidae	2013	1
681	KJ190886	KL21/SD/CHN/10	PrM	650	China	Culicidae	2013	1
682	KJ190887	KL23/SD/CHN/10	PrM	650	China	Culicidae	2013	1
683	KJ190888	KL24/SD/CHN/10	PrM	650	China	Culicidae	2013	1
684	KJ190889	KL29/SD/CHN/10	PrM	650	China	Culicidae	2013	1
685	KJ190890	KL30/SD/CHN/10	PrM	650	China	Culicidae	2013	1
686	KJ190891	KL44/SD/CHN/10	PrM	650	China	Culicidae	2013	1
687	KJ190892	KL47/SD/CHN/10	PrM	650	China	Culicidae	2013	1
688	KJ190893	KL59/SD/CHN/10	PrM	650	China	Culicidae	2013	1
689	KJ190894	KL67/SD/CHN/10	PrM	650	China	Culicidae	2013	1
690	KJ190895	KL68/SD/CHN/10	PrM	650	China	Culicidae	2013	1
691	KJ190896	KL75/SD/CHN/10	PrM	650	China	Culicidae	2013	1

692	KJ190897	KL78/SD/CHN/10	PrM	650	China	Culicidae	2013	1
693	KJ190898	KL82/SD/CHN/10	PrM	650	China	Culicidae	2013	1
694	KJ190899	KL84/SD/CHN/10	PrM	650	China	Culicidae	2013	1
695	KJ190900	KL86/SD/CHN/10	PrM	650	China	Culicidae	2013	1
696	KJ190901	KL87/SD/CHN/10	PrM	650	China	Culicidae	2013	1
697	KJ190902	KL88/SD/CHN/10	PrM	650	China	Culicidae	2013	1
698	KJ190903	KL91/SD/CHN/10	PrM	650	China	Culicidae	2013	1
699	KJ190904	KL92/SD/CHN/10	PrM	650	China	Culicidae	2013	1
700	KJ190905	KL93/SD/CHN/10	PrM	650	China	Culicidae	2013	1
701	KJ190906	KL95/SD/CHN/10	PrM	650	China	Culicidae	2013	1
702	KJ190907	KL97/SD/CHN/10	PrM	650	China	Culicidae	2013	1
703	KJ190908	KL109/SD/CHN/10	PrM	650	China	Culicidae	2013	1
704	KJ190909	KL113/SD/CHN/10	PrM	650	China	Culicidae	2013	1
705	KJ190910	KL114/SD/CHN/10	PrM	650	China	Culicidae	2013	1
706	KJ190911	KL115/SD/CHN/10	PrM	650	China	Culicidae	2013	1
707	KJ190912	KL116/SD/CHN/10	PrM	650	China	Culicidae	2013	1
708	KJ190913	KL119/SD/CHN/10	PrM	650	China	Culicidae	2013	1
709	KJ190914	rc14/SD/CHN/10	PrM	650	China	Culicidae	2013	1
710	KJ190915	rc16/SD/CHN/10	PrM	650	China	Culicidae	2013	1
711	KJ190916	rc20/SD/CHN/10	PrM	650	China	Culicidae	2013	1
712	KJ190917	rc21/SD/CHN/10	PrM	650	China	Culicidae	2013	1
713	KJ190918	rc34/SD/CHN/10	PrM	650	China	Culicidae	2013	1
714	KJ190919	rc35/SD/CHN/10	PrM	650	China	Culicidae	2013	1
715	KJ190920	rc47/SD/CHN/10	PrM	650	China	Culicidae	2013	1
716	KJ190921	rc48/SD/CHN/10	PrM	650	China	Culicidae	2013	1
717	KJ190922	rc49/SD/CHN/10	PrM	650	China	Culicidae	2013	1
718	KJ190923	rc52/SD/CHN/10	PrM	650	China	Culicidae	2013	1
719	KJ190924	rc53/SD/CHN/10	PrM	650	China	Culicidae	2013	1
720	KJ190925	rc54/SD/CHN/10	PrM	650	China	Culicidae	2013	1
721	KJ190926	rc55/SD/CHN/10	PrM	650	China	Culicidae	2013	1
722	KJ190927	VN5/SD/CHN/10	PrM	650	China	Culicidae	2013	1
723	KJ190928	VN10/SD/CHN/10	PrM	650	China	Culicidae	2013	1
724	KJ190929	VN11/SD/CHN/10	PrM	650	China	Culicidae	2013	1
725	KJ190930	VN16/SD/CHN/10	PrM	650	China	Culicidae	2013	1
726	KJ190931	VN21/SD/CHN/10	PrM	650	China	Culicidae	2013	1
727	KJ190932	VN22/SD/CHN/10	PrM	650	China	Culicidae	2013	1
728	KJ190933	VN32/SD/CHN/10	PrM	650	China	Culicidae	2013	1
729	KJ190934	VN35/SD/CHN/10	PrM	650	China	Culicidae	2013	1
730	KJ190935	VN37/SD/CHN/10	PrM	650	China	Culicidae	2013	1
731	KJ190936	VN38/SD/CHN/10	PrM	650	China	Culicidae	2013	1
732	KJ190937	VN47/SD/CHN/10	PrM	650	China	Culicidae	2013	1
733	KJ190938	VN48/SD/CHN/10	PrM	650	China	Culicidae	2013	1
734	KU746972	YS131115	PrM	672	South Korea	Culex pipiens	2013	5
735	MG673540	PD3G_9-S-Cut-R-10-3	PrM	678	China: Songjiang Dist., Shanghai	Culex tritaeniorhynchus	2016	1
736	MN579050	YUN2018A	prm	240	China: Yunnan Province	Culex tritaeniorhynchus	2018	1
737	MN579051	YUN2018B	prm	240	China: Yunnan Province	Culex tritaeniorhynchus	2018	1
738	AB981185	JEV/MQ/Yamaguchi/2013-3	prme	2224	Japan: Yamaguchi, Yoshida	Culex tritaeniorhynchus	2013	1
739	LC143633	Japanese encephalitis virus LC143633	PrM-E	563	Viet Nam	Sus scrofa	2015	1
740	KX033859	VNUA-Vetlab02	prm-e	563	Viet Nam: Thai Binh	Sus scrofa	2015	1
741	MH193524	YN2017-2	PrM-E	807	China: Yunnan province	Culicidae	2017	1
742	LC644192	JEV/Mos/Chiba/24/2020	prm-e	489	Japan	Culex tritaeniorhynchus	2020	1
743	LC644193	JEV/Sw/Chiba/13/2020	prm-e	496	Japan	Sus scrofa	2020	1

Table S2. G1 JEV isolates by region from 2012 to 2022.

G1	China	Chinese Taiwan	Cam-bodia	Indone-sia	India	Japan	Singa-pore	The Republic of Korea	Thai-land	Viet Nam	Subto-tal
2012	31	12				7					50
2013	116		1		5	8		10		1	141
2014	10		2			2	1			1	16
2015	29		8		2	1		1		2	43
2016	40	2			1	2				1	46
2017	78	10		1	1	3		1	1		95
2018	89	8			2	1					100
2019	34	6				4	2				46
2020	1					5	1				7
2021	6					2					8
2022	6	1									7
2012-2022	440 (78.71%)	39 (6.98%)	11 (1.97%)	1 (0.18%)	11 (1.97%)	35 (6.26%)	4 (0.72%)	12 (2.15%)	1 (0.18%)	5 (0.89%)	559

Table S3. G3 JEV isolates by region from 2012 to 2022.

G3	Angola	China	Chinese Taiwan	India	Japan	Philippines	Thailand	Subtotal
2012		1	1	5				7
2013		3		11				14
2014		1		7				8
2015		3		9				12
2016	1	27		3				31
2017		1		2				3
2018		6		30		2		38
2019				7	2		2	11
2020		19						19
2021				6				6
2022								0
2012-2022	1 (0.67%)	61 (40.94%)	1 (0.67%)	80 (53.69%)	2 (1.34%)	2 (1.34%)	2 (1.34%)	149

Table S4. G1 and G3 historical (1930–2022) virus information.

Genotype	Country（Region）	1930–2011	2012–2022
G1	Australia	6 (1.32%)	0
	China	97 (21.37%)	440 (78.71%)
	Chinese Taiwan	39 (8.59%)	39 (6.98%)
	Cambodia	3 (0.66%)	11 (1.97%)
	Indonesia	0	1 (0.18%)
	India	29 (6.39%)	11 (1.97%)
	Japan	164 (36.12%)	35 (6.26%)
	Laos	4 (0.88%)	0
	Malaysia	4 (0.88%)	0
	Singapore	0	4 (0.72%)
	The Republic of Korea	58 (12.78%)	12 (2.15%)
	Thailand	34 (7.49%)	1 (0.18%)
	Vietnam	16 (3.52%)	5 (0.89%)
	Subtotal	454	559
G3	Angola		1 (0.67%)
	China	111 (25.28%)	61 (40.94%)
	Chinese Taiwan	80 (18.22%)	1 (0.67%)
	Indonesia	3 (0.68%)	
	India	117 (26.65%)	80 (53.69%)
	Italy	5 (1.14%)	
	Japan	66 (15.03%)	2 (1.34%)
	Malaysia	10 (2.28%)	
	Myanmar	1 (0.23%)	
	Nepal	2 (0.46%)	
	Philippines	4 (0.91%)	2 (1.34%)
	Sri Lanka	3 (0.68%)	
	The Republic of Korea	23 (5.24%)	
	Thailand	1 (0.23%)	2 (1.34%)
	USSR (now defunct, historical reference)	1 (0.23%)	
	Vietnam	12 (2.73%)	
	Subtotal	439	149
